# tRNA Signatures Reveal a Polyphyletic Origin of SAR11 Strains among Alphaproteobacteria

**DOI:** 10.1371/journal.pcbi.1003454

**Published:** 2014-02-27

**Authors:** Katherine C. H. Amrine, Wesley D. Swingley, David H. Ardell

**Affiliations:** Program in Quantitative and Systems Biology, University of California, Merced, Merced, California, United States of America; The Centre for Research and Technology, Hellas, Greece

## Abstract

Molecular phylogenetics and phylogenomics are subject to noise from horizontal gene transfer (HGT) and bias from convergence in macromolecular compositions. Extensive variation in size, structure and base composition of alphaproteobacterial genomes has complicated their phylogenomics, sparking controversy over the origins and closest relatives of the SAR11 strains. SAR11 are highly abundant, cosmopolitan aquatic Alphaproteobacteria with streamlined, A+T-biased genomes. A dominant view holds that SAR11 are monophyletic and related to both Rickettsiales and the ancestor of mitochondria. Other studies dispute this, finding evidence of a polyphyletic origin of SAR11 with most strains distantly related to Rickettsiales. Although careful evolutionary modeling can reduce bias and noise in phylogenomic inference, entirely different approaches may be useful to extract robust phylogenetic signals from genomes. Here we develop simple phyloclassifiers from bioinformatically derived tRNA Class-Informative Features (CIFs), features predicted to target tRNAs for specific interactions within the tRNA interaction network. Our tRNA CIF-based model robustly and accurately classifies alphaproteobacterial genomes into one of seven undisputed monophyletic orders or families, despite great variability in tRNA gene complement sizes and base compositions. Our model robustly rejects monophyly of SAR11, classifying all but one strain as Rhizobiales with strong statistical support. Yet remarkably, conventional phylogenetic analysis of tRNAs classifies all SAR11 strains identically as Rickettsiales. We attribute this discrepancy to convergence of SAR11 and Rickettsiales tRNA base compositions. Thus, tRNA CIFs appear more robust to compositional convergence than tRNA sequences generally. Our results suggest that tRNA-CIF-based phyloclassification is robust to HGT of components of the tRNA interaction network, such as aminoacyl-tRNA synthetases. We explain why tRNAs are especially advantageous for prediction of traits governing macromolecular interactions from genomic data, and why such traits may be advantageous in the search for robust signals to address difficult problems in classification and phylogeny.

## Author Summary

If gene products work well in the networks of foreign cells, their genes may transfer horizontally between unrelated genomes. What factors dictate the ability to integrate into foreign networks? Different RNAs and proteins must interact specifically in order to function well as a system. For example, tRNA functions are determined by the interactions they have with other macromolecules. We have developed ways to predict, from genomic data alone, how tRNAs distinguish themselves to their specific interaction partners. Here, as proof of concept, we built a robust computational model from these bioinformatic predictions in seven lineages of Alphaproteobacteria. We validated our model by classifying hundreds of diverse alphaproteobacterial taxa and tested it on eight strains of SAR11, a phylogenetically controversial group that is highly abundant in the world's oceans. We found that different strains of SAR11 are more distantly related, both to each other and to mitochondria, than widely believed. We explain conflicting results about SAR11 as an artifact of bias created by the variability in base contents of alphaproteobacterial genomes. While this bias affects tRNAs too, our classifier appears unexpectedly robust to it. More broadly, our results suggest that traits governing macromolecular interactions may be more faithfully vertically inherited than the macromolecules themselves.

## Introduction

Which parts of genomes are most resistant to compositional convergence? Which information is vertically inherited most faithfully? Compositional stationarity and vertical (co-)inheritance are key, yet frequently violated, assumptions of most current approaches in molecular phylogenetics and phylogenomics [1]. Horizontal gene transfer (HGT), for example, is so common and widespread that the very existence of a “Tree of Life” has been called into question [2], [3]. Advances in understanding the history of life will require discovery of new universal, slowly-evolving phylogenetic markers that are resistant to compositional convergence and HGT.

The controversial phylogeny of *Ca.* Pelagibacter ubique (SAR11) is a case in point. SAR11 make up between a fifth and a third of the bacterial biomass in marine and freshwater ecosystems [4]. SAR11 have very small cell sizes, genome sizes, and intergenic region sizes, possibly in adaptation to extreme nutrient limitations [5]. Some recent phylogenomic studies place free-living SAR11 together in a clade with the largely endoparasitic Rickettsiales and the alphaproteobacterial ancestor of mitochondria [6], [7], [8]. Other studies persuasively argue that this placement is an artifact of independent convergence of SAR11 and Rickettsiales towards increased genomic A+T contents, and that SAR11 are more closely related to the free-living Alphaproteobacteria such as the Rhizobiales and Rhodobacteraceae [9],[10],[11]. The monophyly of SAR11 was also recently rejected [10], [12].

Nonstationary macromolecular compositions are a known source of bias in phylogenomics [13], . Widespread variation in macromolecular compositions may be caused by loss of DNA repair pathways in reduced genomes [15], [11], unveiling an inherent A+T-bias of mutation in bacteria [16] that elevates genomic A+T contents [17], [18]. A process such as this has likely altered protein and RNA compositions genome-wide in SAR11, and if such effects are accounted for, SAR11 appear more closely related to Rhizobiales and Rhodobacteraceae than Rickettsiales [10], [11]. Consistent with this interpretation, SAR11 strain HTTC1062 shares, with a large clade of free-living Alphaproteobacteria that excludes the Rickettsiales, a unique and derived codivergence of features that govern recognition between tRNAHis and histidyl-tRNA synthetase (HisRS) [19], [20]. This unique functionally significant synapomorphy likely arose only once in bacteria [21] and independently contradicts affiliation of SAR11 with Rickettsiales.

Can the features that govern interactions between macromolecules improve phylogenomic inferences? The two main phylogenomic “supermatrix” and “supertree” approaches [22] treat homologous sites or genes, respectively, as statistically independent data. Yet gene product interactions have known influences on their evolution. For example, amino acid substitution rates vary inversely with interaction degree (number of interaction partners) in proteins [23]. Furthermore, “informational” classes of genes, which mediate the expression and regulation of other genes, have more direct and indirect interaction partners on average than induced, metabolic “operational” classes of genes [24] and are less frequently exchanged across species by HGT [25], [26]. A celebrated exception to this “complexity hypothesis” — an exception thought to prove the rule — is that of aminoacyl-tRNA synthetases (aaRSs), which are “informational” housekeeping genes with high rates of HGT; this is explained because aaRSs are thought to interact primarily with only one set of tRNA isoacceptor types [27], [28], [29], [30], [31]. Although aaRSs and also tRNAs [32] can have high rates of HGT, the co-evolved features or “rules” that govern their interactions are thought to be quite resistant to lateral transfer [33]. Generally, we propose that laterally acquired gene products are more likely to adapt to new resident networks rather than to remodel those networks in accommodation of themselves.

**Figure 1:**
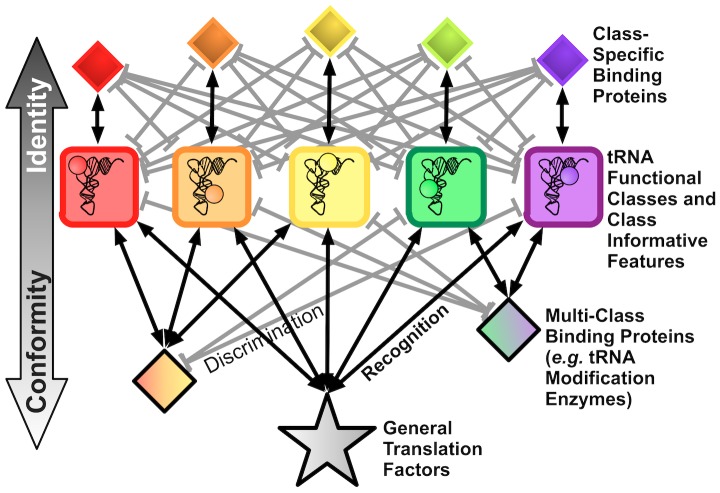
A universal schema for tRNA interaction networks. tRNAs interact to varying degrees of specificity within a strongly conserved network of protein and RNA complexes. The simultaneous and conflicting requirements of “identity” and “conformity” on tRNAs create potential deleterious pleiotropic effects when components of the network mutate or are transferred to foreign cells by HGT. They also facilitate the bioinformatic prediction of Class-Informative Features (CIFs) from tRNAs that function together in the same or similar networks.

Comprehensive, accurate identification and homology mapping of features that govern macromolecular interactions remains challenging in general. tRNAs bring two distinct advantages to such an enterprise. First, the components and interactions in the tRNA interaction network are relatively highly conserved. Second and more importantly, as illustrated in Figure 1, because all tRNAs are globally connected through general translation factors, their structures are highly conserved not only across species but also across different functional varieties of tRNAs (“conformity” [34]). Each functional variety or “class” of tRNA, defined in part by which amino acid it is charged with, is distinguished by increasingly class-specific interactions with tRNA-binding proteins and other factors (“identity” [35]). The uniquely contradictory requirements on tRNAs of conformity and identity makes it possible to predict the features that govern tRNA interactions by relatively simple bioinformatic analysis of genomic tRNA sequence data alone [20].

**Figure 2:**
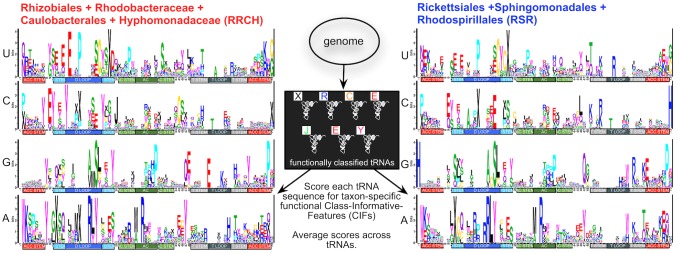
Function logos of structurally aligned tRNA data as calculated by LOGOFUN [36] for two groups of Alphaproteobacteria and overview of tRNA-CIF-based binary phyloclassification. Function logos generalize sequence logos. They are the sole means by which we predict tRNA Class-Informative Features (CIFs), which form the basis of the scoring schemes of the classifiers reported in this work. A full derivation of the mathematics of function logos is provided in [36]. The tRNA-CIF-based phyloclassifier shown in Figure 3A sums differences in heights of features between two function logos for a set of genomically derived tRNAs. Complete source code and data to reproduce the function logos in this figure are in Dataset S1.

In earlier work, we developed “function logos” to predict, at the level of individual nucleotides before post-transcriptional modification, which features in tRNA gene sequences are associated to specific functional classes of tRNAs [36]. More precisely,“class” refers to a functional variety of tRNA (such as amino acid charging or initiator identity). We now call our function-logo-based predictions Class-Informative Features (CIFs). A tRNA CIF answers the question: “If a tRNA gene from a group of related genomes carries a specific nucleotide at a specific structural position, how informative is that feature about function, and how over-represented is that feature in a specific functional class?” Our estimates are corrected for biased sampling of tRNA functional classes and sample size effects [36], and we can calculate their statistical significance [20]. In more practical terms, a tRNA CIF corresponds exactly to a single letter in the types of tRNA function logos shown in Figure 2 in the Results presented below. The “height” or fractional information of such a letter, measured in bits, is the product of conditional information of the feature about function and the normalized odds ratio of its appearance in a particular class. Thus, the greater height such a letter has, the more functionally informative that feature is, and the more it is specifically associated to a particular tRNA functional class above background expectations. We have shown that these traits, already known to have diverged across the three domains of life [37] have evolved and diverged extensively among bacteria [21], [38].

While a single bacterial genome does not present enough tRNA sequence data to generate a statistically significant function logo, data from related genomes may be lumped together. Although this procedure assumes homogeneity, in practice features shared across taxa yield the largest signals, while phyletic variation in class-associations of features reduces signal. Function logos recover known tRNA identity elements (*i.e.* features that govern specific tRNA-aaRS interactions) [37], [35], and more generally, predict features governing interactions with class-specific network partners such as amidotransferases [39]. A recent molecular dynamics study on a tRNA^Glu^ -GluRS (Glutaminal tRNA-synthetase) complex identified functional sites in tRNA^Glu^ involved in allosteric signaling that couple substrate recognition to reaction catalysis in the complex [40]. The predicted sites are associated with those from proteobacterial function logos [38]. Thus, tRNA CIFs predict class-specific functional features beyond strictly tRNA identity elements alone.

In this work, we show that tRNA CIFs have diverged among Alphaproteobacteria in a phylogenetically informative manner, enabling their use as signatures for classification. We validate our approach on diverse alphaproteobacterial genomes. We show that, as with other phylogenetic markers [10],[11], tRNAs in SAR11 and Rickettsiales have converged in base compositions, inducing an artifactual affinity between these groups when more conventional phylogenomic methods are applied to whole tRNA sequences. Our results confirm those of multiple studies that control for genomic base content variation across Alphaproteobacteria, showing that SAR11 is not a clade [10], [12], and that no SAR11 strains have Rickettsiales as their closest relatives [10], [11]. Thus, tRNA CIFs are more robust to compositional convergence than the tRNA bodies in which they are embedded. Our results suggest that the best signals in genomes for deep phylogenetic problems may lie among the features that govern macromolecular interactions.

## Results

In order to characterize tRNA CIFs within Alphaproteobacteria, we reannotated alphaproteobacterial tDNA data from tRNAdb-CE 2011 [41] and pre-publication genomic data for SAR11. For our initial studies, we set aside the SAR11 data and organized our alphaproteobacterial tDNA database taxonomically into two parts, according to whether or not source genomes contained the uniquely derived synapomorphic tRNA^His^ traits described previously [21], [19], [20]. One part corresponded to a phylogenetically coherent “RRCH clade,” comprising the Rhodobacteraceae, Rhizobiales, Caulobacterales, and Hyphomonadaceae, which presented the derived tRNA^His^ traits A73 and absence of the otherwise universally conserved genetically templated 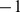
G (defined according to the so-called “Sprinzl coordinates,” standard in the field for enumerating tRNA structural sites [42]). The other part corresponded to an “RSR grade” comprising the Rhodospirillales, Sphingomonadales, and Rickettsiales, which presented “normal” bacterial tRNA^His^ traits C73 and genomically templated 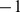
G (an “evolutionary grade” is an ancestral and paraphyletic grouping). Importantly, the RRCH and RSR split defined by tRNA^His^ traits are broadly consistent with all phylogenomic treatments of alphaproteobacterial phylogeny to date [43], [6], [44], [7], [8], [9], [10], [11]. In all, we analyzed 214 alphaproteobacterial genomes presenting 11644 predicted tRNA gene sequences (8773 sequences unique within their respective genomes and 3064 sequences unique overall). Our RRCH clade data comprised 8597 tRNA genes from 147 genomes, while our RSR grade data comprised 2792 tRNA genes from 59 genomes. We analyzed 255 tRNA genes from eight SAR11 strain genomes.

Seven of eight SAR11 strain genomes available to us exhibited the unique tRNA^His^/HisRS codivergence traits in common with RRCH clade genomes. In contrast, strain HIMB59 presented ancestral bacterial characters in both tRNA^His^ and HisRS in common with the RSR grade genomes (tRNA data not shown, HisRS data shown in Figure S1). These results immediately suggested, consistent with [10] and [12], that HIMB59 is not monophyletic with the other SAR11 strains and is affiliated with the RSR grade, while most other SAR11 strains are unrelated to the Rickettsiales and belong in the RRCH clade.

In previous work, we reported the existence of fairly extensive and general divergence of tRNA Class-Informative Features (CIFs) between Proteobacteria and Cyanobacteria [38]. In order to investigate tRNA CIF divergence within the Alphaproteobacteria, we computed function logos [36] of the RRCH clade and RSR grade tDNA data. Qualitatively, the RRCH and RSR function logos provide visible evidence of general tRNA CIF divergence between these two groups (comparing function logos in Figure 2). To quantify these differences and exploit them to classify genomes, we formulated a quantitative measure of how well tRNAs from a given alphaproteobacterial genome match the tRNA CIFs of one group or another. Our initial simple scoring scheme sums up the differences in fractional information values or heights of features in two different function logos for two taxonomic groups if tRNAs of a given genome of the correct class carry those features (see Figure 2 and Materials and Methods). To reduce bias, we used a Leave-One-Out Cross-Validation (LOOCV) approach, in which we recomputed the RRCH or RSR function logos for each genome to be classified by removing its own contribution to the data. In order to compare the results against those that we would get using the entire tRNA sequences, we also scored genomes using the sum of log-odds of entire sequences from tRNA-class-specific RRCH and RSR tRNA sequence profiles, also with an LOOCV approach.

**Figure 3:**
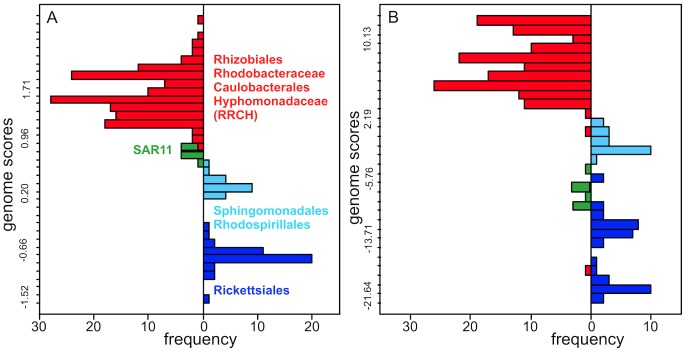
Leave-One-Out Cross-Validation (LOO-CV) scores of alphaproteobacterial genomes under two different binary phyloclassifiers. A. Score distribution of genomes under the binary tRNA-CIF-based phyloclassifier as sketched in Figure 2. The score of a genome in this classifier is the summation of differences in heights of the features of its tRNAs in the RRCH and RSR function logos in Figure 2. B. Scores under the “zero” total tRNA sequence-based phyloclassifer defined in Materials and Methods and conducted as a control. Here the score of a genome is just the sum of log-odds of its tRNA sequences in two class-specific sequence profiles from the RRCH and RSR clades. See Figure S2 for alternative treatments of missing data under other methods. Complete source code and data to reproduce these results and those in Figure S2 are in Dataset S2.

Typical results are shown in Figure 3. Although the tRNA-CIF-based phyloclassifier (Figure 3A) was biased positively by the much larger RRCH sample size, it achieved better phylogenetic separation of genomes than the total-tRNA-sequence-based phyloclassifier based on taxon-specific tRNA profiles for different functional classes (Figure 3B). The Sphingomonadales and Rhodospirillales separated in scores from the Rickettsiales in both classifiers. Most importantly, the tRNA-CIF-based phyloclassifier placed all eight SAR11 genomes closer to the RRCH clade and far away from the Rickettsiales with HIMB59 overlapping the Rhodospirillales, while the total-tRNA-sequence-based phyloclassifier placed all eight SAR11 genomes closer to the Rickettsiales. Overall, while both scoring schemes separated taxonomically distinct clades, these results show that CIFs and total tRNA data yield different signals regarding the phylogenetic placement of SAR11 genomes. Figure S2 shows the effects of different treatments of missing data in the total-tRNA-sequence-based classifier. Method “zero,” shown in Figure 3B, is most analogous to the method used to generate Figure 3A. Method “skip” (Figure S2B) shows that SAR11 tRNAs share sequence characters in common with the RSR grade that are not seen in the RRCH clade. Methods “small” and “pseudo” (Figures S2C and S2D) show that SAR11 have sequence traits not observed in either the RSR or RRCH datasets.

**Figure 4:**
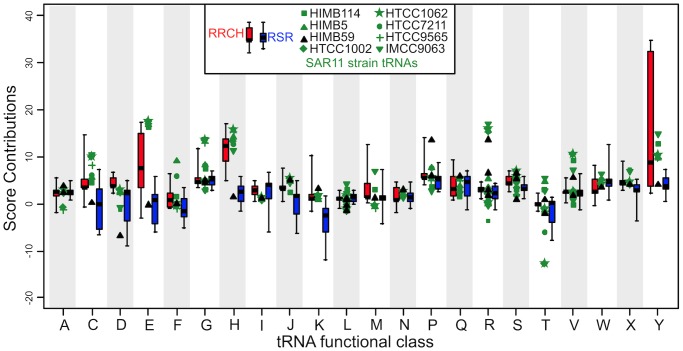
Breakout of class contributions to scores under the tRNA CIF-based binary phyloclassifier. Contributions of each functional variety of tRNA, or class, to the tRNA-CIF-based phyloclassifier scores in Figure 3A. Different SAR11 strain tRNAs are plotted separately by genome of origin. Complete source code and data to reproduce these results are in Dataset S3.

Divergence of tRNA CIFs between the RRCH clade and RSR grade is general and encompasses other classes besides tRNA^His^. Other classes that contributed strongly to differentiated classification of RRCH and RSR genomes by the tRNA CIF-based binary classifier include tRNA^Cys^, tRNA^Asp^, tRNA^Glu^, 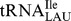 (symbolized “J”), tRNA^Lys^, and tRNA^Tyr^ (Figure 4). In a manual curation of the most obvious CIF differences between RRCH and RSR, we identified traits specific to RRCH including C7-Tyr, R8-Tyr and U15∶G48-Glu, all with heights greater than 2 bits (the height of a CIF is the height of its letter in a function logo as shown in Figure 2, which specifically quantifies both functional information and over-representation of a CIF in tRNAs of a particular functional class and taxonomic group; please see Materials and Methods and [45], [36] for more details). RSR-specific CIFs include A12-Cys and C52∶G62-Lys. These results extend the observations of [19] who discovered unusual base-pair features of tRNAGlu among members of the RRCH clade. Also, our results suggest that the unique codivergence caused by HGT of a eukaryotic-derived HisRS into an ancestor of the RRCH clade has perturbed interactions in other tRNAs, in keeping with their network coupling as shown in Figure 1. In classes for which the RRCH and RSR groups are well-differentiated, SAR11 strain HIMB59 uniquely groups with RSR while other SAR11 strains group with RRCH, while for other tRNA classes, all putative SAR11 strains lie outside the RRCH and RSR distributions. These results imply that more diverse alphaproteobacterial genomic data are necessary to completely resolve the phylogenetic affiliation of SAR11 strains, but strongly contradict a monophyletic affiliation of SAR11 with Rickettsiales.

**Figure 5:**
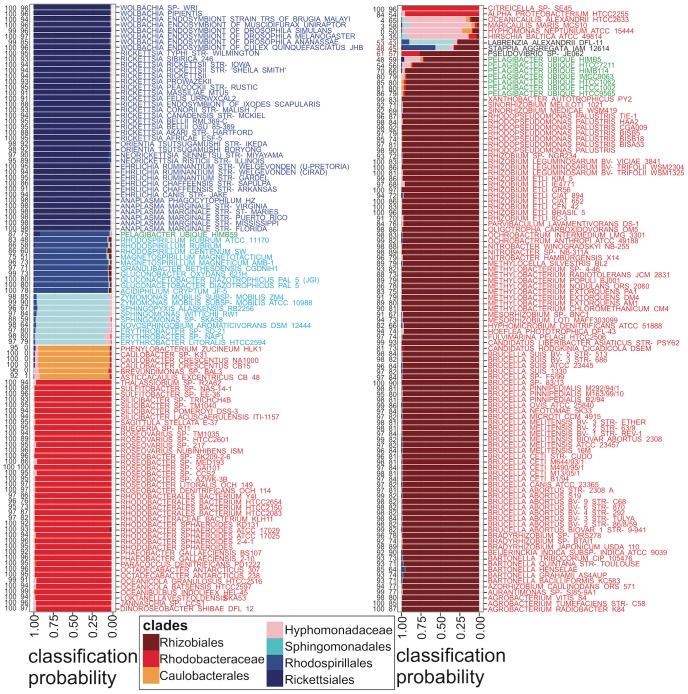
Seven-way tRNA-CIF-based phyloclassification of alphaproteobacterial genomes by the default multilayer perceptron in WEKA. Each test genome classified is assigned a probability of classification into each of the seven alphaproteobacterial clades indicated. Bootstrap support values under resampling of tRNA sites against (left) all tRNA CIFs and (right) CIFs with heights 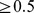 bits and model retraining (100 replicates). All support values correspond to most probable clade as shown except for *Stappia* and *Labrenzia* for which they correspond to Rhizobiales. Complete source code and data to produce this figure, including the full WEKA model for classification of other alphaproteobacterial genomes and code to produce such models from scratch, is provided in Dataset S4.

In order to expand on this preliminary binary classification, we developed a multiway tRNA CIF-based classifier for alphaproteobacterial genomes. Instead of computing a simple difference of summed scores as before, the multiway classifier uses seven scores as its input features, in which each score sums evidence that tRNAs from a query genome match the tRNA CIFs of a specific subclade of Alphaproteobacteria. We used these summed scores to train the default multilayer perceptron (MLP) model implemented in WEKA [46] with ten-fold cross-validation to avoid overfitting. The MLP is the simplest nonlinear classifier able to handle the phylogenetically dependent signals in our score vectors [47]. The output of the MLP is a seven-element vector giving the classification probabilities of the query genome for each of the seven clades. Again using an LOOCV approach, each genome in our dataset classified consistently with published taxonomic positions [6], [44], [8], [9], [10], [11] as expressed through NCBI Taxonomy, except for all eight SAR11 strains and three additional taxa recently placed in the Rhodobacteraceae based on 16S ribosomal RNA evidence: *Stappia aggregata* [48], *Labrenzia alexandrii* [49] and the denitrifying *Pseudovibrio sp.* JE062 [50] (Figure 5). Our results for SAR11 are exactly consistent with those of [10]: all SAR11 strains except HIMB59 classify as Rhizobiales, while strain HIMB59 classifies as Rhodospirillales. Furthermore, *Stappia*, *Labrenzia* and *Pseudovibrio* classify poorly or not at all as Rhodobacteraceae. *Pseudovibrio* classified four times more strongly as Rhizobiales than as Rhodobacteraceae.

Even excluding SAR11, the alphaproteobacterial genomes that we analyzed vary remarkably in both tRNA gene numbers (reflecting genome size variation) and tRNA G+C contents. Genomic tRNA numbers vary from under 20 for highly reduced endosymbiotic genomes to over 110, while tRNA G+C contents range from about 53% for some Rickettsiales to over 62% for *Methylobacterium* and *Magnetospirillum* (Table S1). Despite this variation, most classifications in Figure 5 were strongly and consistently statistically supported, indicating that our classifier is generally robust to base content variation of tRNAs and even deletion of entire tRNA classes. In two different bootstrap analyses, we bootstrapped sites of tRNA data in each genome to be classified, and we also filtered away small CIFs with heights 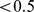 bits from our models, retrained the classifier and bootstrapped sites again. Generally, the majority of bootstrap classifications matched the original dominant classifications. Alphaproteobacteria with more A+T-rich tRNAs such as members of the genus *Ehrlichia* classified correctly in order Rickettsiales with high probability and bootstrap values of 100 (or an average of 92.5 using only CIFs with heights above 0.5 bits). At the other extreme with more G+C-rich tRNAs in the genus *Methylobacteria*, all strains classified correctly as Rhizobiales with a mean bootstrap value of 89 (or 78 using only CIFs with heights above 0.5 bits). *Azorhizobium caulinodans*, belonging in the Rhizobiales, has G+C-rich tRNAs at 62%, and is the only representative of its genus in our study. Even in a Leave-One-Out Cross-Validation, *A. caulinodans* classified correctly with bootstrap values of 94 and 77, respectively.

In our CIF bootstrap analyses, SAR11 strains either had support values greater than 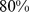 as Rhizobiales, majority bootstrap values as Rhizobiales (HIMB114 at 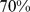 with Rickettsiales at 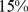 and HTCC7211 at 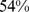 with Rickettsiales at 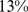), or a plurality bootstrap value as Rhizobiales (HIMB5 at 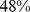 with Rickettsiales at 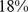), except for HIMB59 which had a bootstrap support value of 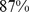 as Rhodospirillales. Full bootstrap statistics over all seven clades with these models are provided in Table S2 for SAR11, *Stappia*, *Labrenzia* and *Pseudovibrio*. In a separate analysis, we also deleted each one of the 22 functional tRNA classes from the data training multiway classification (Table S3). Classification results for all of the “known” training genomes were generally highly stable to the deletion of a tRNA functional class, with a maximum of only six out of 203 genomes changing taxonomic classifications upon deletion of any one of the following tRNA functional classes: Cys, His, Arg, and Gly.

**Figure 6:**
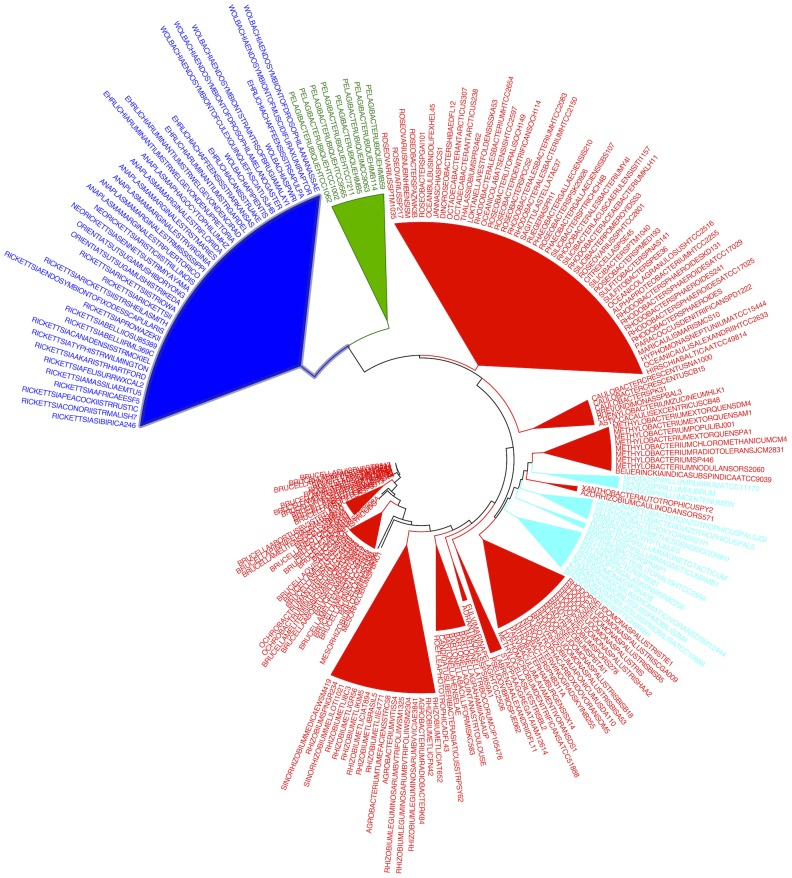
FastUniFrac-based phylogenetic tree of alphaproteobacteria using tRNA data computed according to the methods of [51]. The FastUniFrac algorithm was recently adapted as a phylogenomic method using tRNA genes. Like the supermatrix phylogenomic approach on tRNAs with results shown in Figures S3 and S4, this method uses unfiltered total sequence information of tRNAs. In contrast to Figure 5, both in this figure and in Figures S3 and S4, all SAR11 strains are affiliated with Rickettsiales. For reasons shown in Figure 7, we argue these results are artifacts of convergence in tRNA base contents. Complete source code and data to reproduce these results are in Dataset S5.

When using total tRNA sequence evidence, we could not reconstruct results similar to those in Figure 5, by either a “classical” phylogenomic supermatrix analysis of tRNAs, or using the recent novel FastUnifrac based approach specifically adapted for tRNA data [51]. In a “supermatrix” phylogenomic approach, concatenating genes for 28 isoacceptor tRNA classes from 169 species (2156 total sites) and using the GTR+Gamma model in RAxML, we estimated a Maximum Likelihood tree in which all eight putative SAR11 strains branch together with Rickettsiales (Figure S3). For this analysis, in 31% of instances when isoacceptor genes were picked from a genome, we randomly picked one gene from a set of isoacceptor paralogs. However, our results did not depend on which paralog we picked. Using a distance-based approach with FastTree, we computed a consensus cladogram over 100 replicate alignments each representing different randomized picks over paralogs. As shown in a consensus cladogram (Figure S4) each replicate distance tree placed all eight putative SAR11 strains together with the Rickettsiales. Widmann Et Al. (2010) [51] introduced a novel phylogenomic approach that computes a distance tree of all tRNA sequences from all genomes, and then clusters genomes using the UniFrac metric applied to that tree. Their method, although innovative, is also based on total tRNA sequence evidence. We found that it also places all SAR11 strains together with Rickettsiales (Figure 6). These results strengthen those shown in Figures 3 and S2 which suggest that tRNA CIFs exhibit a specific evolutionary signal distinct from that of tRNA sequences as a whole.

**Figure 7:**
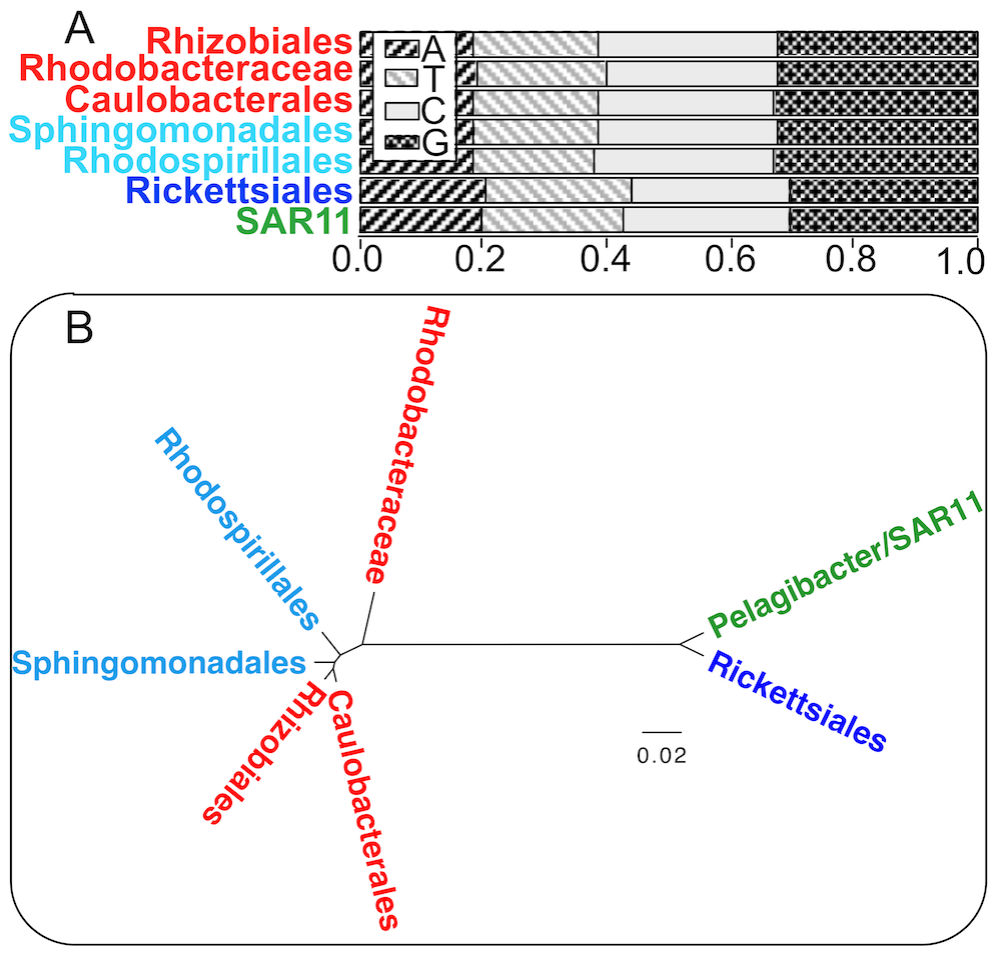
Base compositions of alphaproteobacterial tRNAs showing convergence between Rickettsiales and SAR11. A. Stacked bar graphs of tRNA base compositions by clade. B. UPGMA clustering of clades based on Euclidean distances of tRNA base compositions under the centered log ratio transformation [88]. tRNA base compositions alone are sufficient to group all SAR11 strains together with Rickettsiales as a clade. Most popular molecular evolutionary models in use today do not account for base content variation as a source of bias in phylogenetic estimation. Complete source code and data to reproduce these results are in Dataset S6.

Results with total tRNA sequence evidence mirror those with 16S ribosomal RNA [52] in placing all SAR11 strains together with the Rickettsiales. We suspected that it was variability in base contents of alphaproteobacterial tRNAs — caused in part by convergence of SAR11 and Rickettsiales tRNA genes to greater A+T contents — that contributed most greatly to the discrepancies in classification results between our CIF-based classifier and the phylogenomic methods using total tRNA evidence. Increases in genomic A+T in SAR11 and the Rickettsiales have driven increases in A+T content of ribosomal RNA genes [10]. We found evidence of convergence to greater A+T contents of tRNA genes as well (Figure 7A). Rickettsiales and SAR11 tRNA genes are notably elevated in both A and T, and share an overall similarity in compositions distinct from those of other Alphaproteobacteria. Furthermore, a hierarchical clustering of Alphaproteobacterial families and orders based on tRNA gene base contents closely group SAR11 and Rickettsiales together (Figure 7B).

## Discussion

We have exploited our now well-established function logo approach [36], which predicts functional sites in tRNAs, as a means to statistically classify genomes. We have shown that our approach is more robust to tRNA base content variation than more conventional phylogenomic approaches using total tRNA evidence. While our simple scoring schemes are not interpretable as evolutionary distances, in other work we have developed evolutionary distances based on tRNA CIFs and used them to reconstruct phylogenetic trees.

Our results provide strong, albeit unconventional, evidence that most SAR11 strains are affiliated with Rhizobiales, while strain HIMB59 is affiliated with Rhodospirillales. Our results are completely consistent with phylogenomic studies that control for nonstationary macromolecular compositions among Alphaproteobacteria [9], [10], [11], [12] and also with a site-rate-filtered phylogenomic analysis [44]. Our CIF-based method works even though SAR11 tRNAs and Rickettsiales tRNAs have converged in base contents (Figure 7). tRNA CIFs must be at least partly robust to compositional convergence of the tRNA bodies in which they are embedded.

Our results suggest that tRNA-CIF-based phyloclassification is robust to HGT of components of the tRNA interaction network. Our alphaproteobacterial phyloclassifications were highly consistent and showed no signs of misclassification of individual genomes, even though aminoacyl-tRNA synthetases (aaRS) are highly prone to HGT [27], [28], [29], [30], [31] including in the Alphaproteobacteria [21], [53], [54]. tRNAs are also known to be horizontally transferred [32], although confident estimation of tRNA HGT rates is difficult. Even while HGT of tRNAs and tRNA-interacting proteins may be common, HGT of foreign tRNA “identity rules” governing tRNA interactions must be relatively rare. This argument is consistent with that of [33], who argued that a horizontally transferred aaRS is more likely to functionally ameliorate to a tRNA interaction network into which it has been transferred rather than remodel that network to accommodate itself. HGT of components may also perturb a network so as to cause a distinct pattern of divergence [21]. Wang *et al.* [19] discuss the possibility that RRCH tRNAHis and HisRS were co-transferred into an ancestral SAR11 genome. However, this hypothesis fails to explain the correlations of many other tRNA traits of SAR11 genomes with the RRCH clade reported here. Further investigation will be needed to clarify how HGT of aaRSs and tRNAs affect the evolution of tRNA CIFs and our novel phyloclassification method.

A more distant relationship between SAR11 strains and Rickettsiales actually strengthens the genome streamlining hypothesis [5]. With a placement of SAR11 within Rickettsiales, it becomes more difficult to justify how genome reduction in SAR11 occurred by a selection-driven evolutionary process rather than the drift-dominated erosion of genomes in the Rickettsiales [55], [17], [56]. By the same token, polyphyly of nominal SAR11 strains implies that the extensive similarity in genome structure and other traits between HIMB59 and SAR11 reported by [57] may have originated independently. Perhaps convergence in some traits is consistent with selective streamlining, which could also explain trait-sharing between SAR11 and *Prochlorococcus*, marine cyanobacteria also argued to have undergone streamlining [58]. The very clear signs of data limitation evident from results shown in Figures 3, 4, 5 and S2 imply that better taxonomic sampling will improve our results and could ultimately resolve more than two origins of SAR11-type genomes among Alphaproteobacteria.

We extracted accurate and robust phylogenetic signals from tRNA gene sequences by first integrating within genomes to identify features likely to govern functional interactions with other macromolecules. Unlike small molecule interactions, macromolecular interactions are mediated by genetically determined structural and dynamic complementarities. These are intrinsically relative; a large *neutral network* [59] of interaction-determining features should be compatible with the same interaction network. Coevolutionary divergence — turnover—of features that mediate macromolecular interactions, while conserving network architecture, has been described in the transcriptional networks of yeast [60], [61] and worms [62] and in post-translational modifications underlying protein-protein interactions [63]. Coevolutionary divergence of features governing tRNA interactions may be driven by ongoing recruitment of tRNA genes to new functional classes [64]. This work demonstrates that generally, divergence of interaction-governing features is phylogenetically informative.

How features that govern macromolecular interactions diverge is an open question, with possibilities including compensatory nearly neutral mutations [65], fluctuating selection [66], adaptive reversals [67], and functionalization of pre-existent variation [68]. Major changes to interaction interfaces may be sufficient to induce genetic isolation between related lineages, as discussed for the 16S rRNA- and 23S rRNA-based standard model of the “Tree of Life,” in which many important and deep branches associate with large, rare macromolecular changes (“signatures”) in ribosome structure and function [69], [70], [71].

In summary, we propose that tRNA CIFs represent one of many possible different lineage-specific “shape codes” [20] among coinherited macromolecules. The concept of tRNA identity as a “second genetic code” is an old one [72], [73], [74], [75] as recounted in [76]. However, by “shape code” we intend to emphasize the potentially arbitrary and co-evolveable nature of the features that underlie macromolecular interactions in specific lineages. The shape codes of macromolecular interactions within specific cellular lineages not only create a barrier to HGT of components but resist transfer even when HGT of those components occurs. Therefore, the interaction-mediating features of macromolecules may be systems biology's answer to the phylogeny problem. Perhaps no other traits of genomes are vertically inherited more consistently than those that mediate functional interactions with other macromolecules in the same lineage. In fact, the structural and dynamic basis of interaction among macromolecular components — essential to their collaborative function in a system — may define a lineage better than any of those components can themselves, either alone or in ensemble.

## Materials and Methods

Supplementary data packages are provided to reproduce all figures from raw data and enable third-party classification of alphaproteobacterial genomes (Datasets S1, S2, S3, S4, S5, S6, S7, S8).

### tRNA Data

The 2011 release of the tRNAdb-CE database [41] was downloaded on August 24, 2011. From this master database, we selected Alphaproteobacteria data as specified by NCBI Taxonomy data (downloaded September 24, 2010, [77]). Also using NCBI Taxonomy, we further tripartitioned Alphaproteobacterial tRNAdb-CE data into those from the RRCH clade, the RSR grade (excluding SAR11), and three SAR11 genomes, as documented in Supplementary data for figure 2. Five additional SAR11 genomes (for strains HIMB59, HIMB5, HIMB114, IMCC9063 and HTCC9565) were obtained from J. Cameron Thrash courtesy of the lab of S. Giovannoni. We custom annotated tRNA genes in these genomes as the union of predictions from tRNAscan-SE version 1.3.1 (with -B option, [78]) and Aragorn version 1.2.34 [79]. We classified initiator tRNAs and tRNA^Ile^_CAU_ using TFAM version 1.4 [80] using a model previously created to do this based on identifications in [81] provided as supplementary data. We aligned tRNAs with covea version 2.4.4 [82] and the prokaryotic tRNA covariance model [78], removed sites with more than 97% gaps with a bioperl-based utility [83], and edited the alignment manually in Seaview 4.1 [84] to remove CCA tails and remove sequences with unusual secondary structures. We mapped sites to Sprinzl coordinates manually [42] and verified by spot-checks against tRNAdb [85]. We added a gap in the −1 position for all sequences and G-1 for tRNA^His^ in the RSR group [19].

### Analysis of HisRS Data

We reannotated HisRS genes from a custom BLAST database of the eight SAR11 strain genomes using previously identified HisRS inferred protein sequences from SAR11 strains HTCC1002, HTCC1062 and HTCC7211 and IMCC9063 downloaded from NCBI on September 27, 2012. Using tBLASTn from commandline BLAST version 2.2.27+ [86], we found one match to each SAR11 strain genome, extracted these sequences and aligned them using clustalw2 (v 2.0.11) [87].

### tRNA CIF Estimation and Binary Classifiers

Our tRNA-CIF-based binary phyloclassifier with Leave-One-Out Cross-Validation (LOO CV) is computed directly from function logos, estimated from tDNA alignments as described in [36]. Here, we define a *feature* 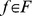 as a nucleotide 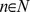 at a position 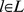 in a structurally aligned tDNA, where 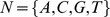 and 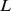 is the set of all Sprinzl coordinates [42]. The set 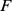 of all possible features is the Cartesian product 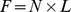. A *functional class* or *class* of a tDNA is denoted 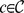 where 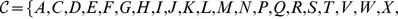 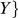 is the universe of functions we here consider, symbolized by IUPAC one-letter amino acid codes (for aminoacylation classes), 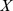 for initiator tRNAs, and 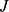 for 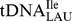. A *taxon set of genomes* or just *taxon set* 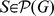 is a set of genomes, where 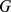 is the set of all genomes, and 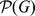 is the power set of 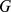. In this work a genome 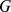 is represented by the multiset of tDNA sequences it contains, denoted 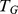. The functional information of features is computed with a map 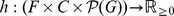 from the Cartesian product of features, classes and taxon sets to non-negative real numbers. For a feature 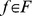, class 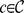 and taxon set 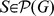, 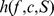 is the fraction of functional information or “height,” measured in bits, associated to that feature, class and taxon set. This height is the product of conditional functional information of a feature (corrected for bias due to sampling), times the normalized odds ratio of it appearing in a specific class [45], see Figure S5 for more detail. In this work, for a given taxon set 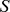, a function logo 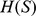 is the tuple:
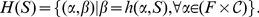
(1)


Furthermore the set 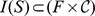 of *tRNA Class-Informative Features* for taxon set 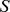 is defined:
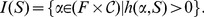
(2)


Briefly, a tRNA Class-Informative Feature is a tRNA structural feature that is informative about the functional classes it associates with, given the context of tRNA structural features that actually co-occur among a taxon set of related cells, and corrected for biased sampling of classes and finite sampling of sequences [36]. Let 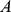 denote a set of Alphaproteobacterial genomes partitioned into three disjoint subsets 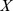, 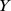 and 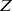 with 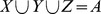, representing genomes from the RRCH clade, the RSR grade, and the eight nominal *Ca.* Pelagibacter strains respectively. To execute the Leave-One-Out Cross-Validation of a tRNA CIF-based binary phyloclassifier for a genome 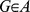 as shown in Figure 3A, we compute a score 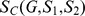, averaging contributions from the multiset 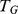 of tDNAs in 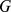 scored against two function logos 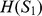 and 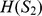 computed respectively from two disjoint taxon sets 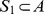 and 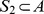, with 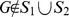. In this study, those sets are 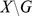 and 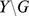, denoted 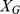 and 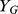 respectively. Each tDNA 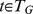 presents a set of features 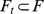 and has a functional class 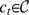 associated to it. The score 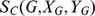 is then defined:
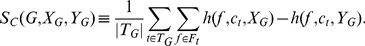
(3)


As controls, we implemented four total-tDNA-sequence based binary phyloclassifiers to score a genome 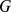, shown in Figures 3B and S2. All are slight variations in which a tRNA 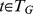 of class 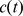 contributes a score that is a difference in log relative frequencies of the features it shares in class-specific profile models generated from 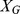 and 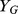. The default “zero” scoring scheme method 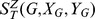 shown in Figure 3B is defined as:
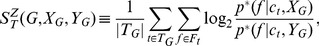
(4)where
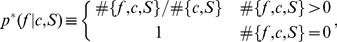
(5)

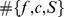
 is the observed frequency of feature 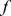 in tDNAs of class 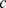 in set 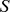, and 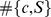 is the frequency of tDNAs of class 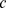 in set 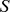.

Method “skip” corresponding to scoring scheme 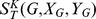 and Figure S2B defined as:
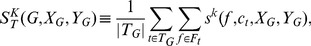
(6)where
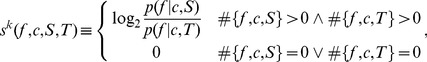
(7)and 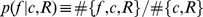 for 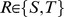 as before.

Methods “pseudo” and “small” corresponding to scoring schemes 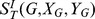 and Figure S2C and S2D respectively:
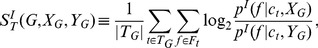
(8)where
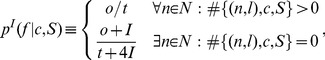
(9)where 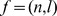, 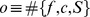, 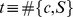, 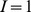 for method “pseudo,” and, for method “small,” 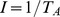, where 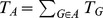.

### Analysis of tRNA Base Composition

To create Figure 7, we computed the base composition of tRNAs aggregated by clades using bioperl-based [83] scripts, and transformed them by the centered log ratio transformation [88] with a custom script provided as supplementary data. We then computed Euclidean distances on the transformed composition data, and performed hierarchical clustering by UPGMA on those distances as implemented in the program NEIGHBOR from Phylip 3.6b [89] and visualized in FigTree v.1.4.

### Supermatrix and FastUniFrac Analysis

For supermatrix approaches, we created concatenated tRNA alignments from 169 Alphaproteobacteria genomes (117 RRCH, 44 RSR, 8 PEL) that all shared the same 28 isoacceptors with 77 sites per gene (2156 total sites). In cases where a species contained more than a single isoacceptor, one was chosen at random. Using a GTR+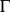 model, we ran RAxML by means of The iPlant Collaborative project RAxML server (http://www.iplantcollaborative.org, [90]) on January 23, 2013 with their installment of RAxML version 7.2.8-Alpha (executable raxmlHPC-SSE3, a sequential version of RAxML optimized for parallelization) (Figure S3). We tested the robustness of our result to random picking of isoacceptors by creating 100 replicate concatenated alignments and running them through FastTree [91] (Figure S4). For the FastUniFrac analysis (Figure 6) we used the FastUniFrac [92] web-server at http://bmf2.colorado.edu/fastunifrac/ to accommodate our large dataset. We removed two genomes from our dataset for containing fewer than 20 tRNAs, and following [51] removed anticodon sites. Following [51] deliberately, we computed an approximate ML tree based on Jukes-Cantor distances using FastTree [91]. We then queried the FastUniFrac webserver with this tree, defining environments to be genomes of origin. We then computed a UPGMA tree based on the server's output FastUniFrac distance matrix in NEIGHBOR from Phylip 3.6b [89].

### Multiway Classifier

All tDNA data from the RSR and RRCH clades were partitioned into one of seven monophyletic clades: orders Rickettsiales (N = 40 genomes), Rhodospirillales (N = 10), Sphingomonadales (N = 9), Rhizobiales (N = 91), and Caulobacterales (N = 6), or families Rhodobacteraceae (N = 43) or Hyphomonadaceae (N = 4) as specified by NCBI taxonomy (downloaded September 24, 2010, [77]) and documented in supplementary data for figure 7. We withheld data from the eight nominal SAR11 strains, as well as from three genera *Stappia*, *Pseudovibrio*, and *Labrenzia*, based on preliminary analysis of tDNA and CIF sequence variation. Following a related strategy as with the binary classifier, we computed, for each genome, seven tRNA-CIF-based scores, one for each of the seven Alphaproteobacterial clades as represented by their function logos, using the principle of Leave-One-Out Cross-Validation (LOO CV), that is, excluding data from the genome to be scored. Function logos were computed for each clade as described in [36]. For each taxon set 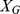 (with genome 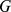 left out if it occurs), genome 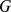 obtains a score 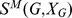 defined by:
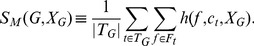
(10)


Each genome 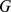 is then represented by a vector of seven scores, one for each taxon set modeled. These labeled vectors were then used to train a multilayer perceptron classifier in WEKA 3.7.7 (downloaded January 24, 2012, [46]) by their defaults through the command-line interface, which include a ten-fold cross-validation procedure. We bootstrap resampled sites in genomic tRNA alignment data (100 replicates) and also bootstrap resampled a reduced (and retrained) model including only CIFs with heights greater than 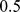 bits.

## Supporting Information

Dataset S1 **Source code and data to reproduce** **Figure 2** **.**
(ZIP)

Dataset S2 **Source code and data to reproduce** **Figures 3** **and S2.**
(ZIP)

Dataset S3 **Source code and data to reproduce** **Figure 4** **.**
(ZIP)

Dataset S4 **Source code and data to reproduce** **Figure 5** **, WEKA model to classify alphaproteobacterial genomes and instructions to extend and generate new WEKA models from tRNA CIF data.**
(ZIP)

Dataset S5 **Source code and data to reproduce** **Figure 6** **.**
(ZIP)

Dataset S6 **Source code and data to reproduce** **Figure 7** **.**
(ZIP)

Dataset S7 **Source code and data to reproduce Figure S1.**
(ZIP)

Dataset S8 **Source code and data to reproduce Figures S3 and S4.**
(ZIP)

Figure S1 **Sequence variation of HisRS motif IIb tRNA-binding loops in SAR11 strains.** Frequency plot logos of the motif IIb tRNA-binding loop of inferred HisRS proteins from putative SAR11 strain genomes. Seven of eight putative SAR11 genomes show the derived characteristic Gly123 unique to the RRCH clade, while one, HIMB59, shows the ancestral Gln123 common to the RSR group and most other bacteria [21], which specifically interacts with the ancestral G-1∶C73 base-pair in tRNA^His^ [93]. These data covary perfectly with tRNA^His^ consistent with affiliation of seven of eight SAR11 strains with the RRCH clade, and of HIMB59 with the RSR grade. Logos made in WebLogo [94].
(EPS)

Figure S2 **Leave-one-out cross-validation scores of alphaproteobacterial genomes under the tRNA sequence-based binary phyloclassifer, using four different methods for handling missing data.** When a genome presents tRNA features missing from one or the other training data sets for the RRCH clade (in red) or RSR grade (in blue). SAR11 data is in green. Method “zero” is shown in the main text as Figure 3B. See Materials and Methods for definitions of “small,” “pseudo” and “skip.”
(EPS)

Figure S3
**Maximum likelihood phylogram of a concatenated supermatrix of 28 isoacceptor genes for 169 alphaproteobacterial genomes computed in RAxML using the GTR+** 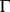 **model.** For genomes in which paralog “isodecoders” of the same isoacceptor gene, one paralog was picked randomly. This occurred in 31% of cases, where a case is one genome x isoacceptor combination. Rickettsiales genomes are boxed in blue and all eight putative SAR11 strains are boxed in green.(EPS)

Figure S4 **Consensus cladogram of 100 replicates of distance-based trees computed in FastTree, each with different randomized picks of isoacceptor genes for alphaproteobacterial genomes in which paralogs for the same isoacceptor exist (also called “isodecoders”).** A. Complete cladogram, with Rickettsiales boxed in blue and putative SAR11 genomes, including HIMB59, in green. B. Magnification showing perfect replicate support for monophyly of Rickettsiales and the eight putative SAR11 strains.
(EPS)

Table S1 **Numbers and base compositions of 214 alphaproteobacterial tRNA genes.** This PDF file has its generating source file and raw data in CSV format attached.
(PDF)

Table S2 **Frequencies out of 100 bootstrap replicates that specific alphaproteobacterial test genomes classified into one among seven alphaproteobacterial clades.** This PDF file has its generating source file and raw data in CSV format attached.
(PDF)

Table S3 **Classifications of 214 alphaproteobacterial genomes across seven alphaproteobacterial clades after deletion of one of 22 different tRNA functional classes using the MLP multiway classifier model in WEKA.** Genomes are ordered to match, top-to-bottom and left-to-right, Figure 5. Clades are symbolized as follows: K, Rickettsiales; D, Rhodospirillales; S, Sphingomonadales; C, Caulobacterales; B, Rhodobacteraceae; H, Hyphomonadaceae; Z, Rhizobiales. For each genome, the 22 clade classfications/functional class deletions are ordered by decreasing robustness of classifications to deletion over all genomes considered known (all but SAR11, *Stappia, Labrenzia and Pseudovibrio*). The class order is as follows: F,T,K,E,L,X,P (203 out of 203 genomes), S (202 genomes), A,I (201 genomes), N,Y,Q,M,J,W (200 genomes) V,D (199 genomes), C,H,R,G (197 genomes). This PDF file has its generating source file and raw data in CSV format attached.
(PDF)
