# Supplementary figures and images for: tRNA Signatures Reveal a Polyphyletic Origin of SAR11 Strains among Alphaproteobacteria
Source: PLoS Comput Biol. 2014 Feb 27;10(2):e1003454. doi: 10.1371/journal.pcbi.1003454 (PMC3937112; doi:10.1371/journal.pcbi.1003454)

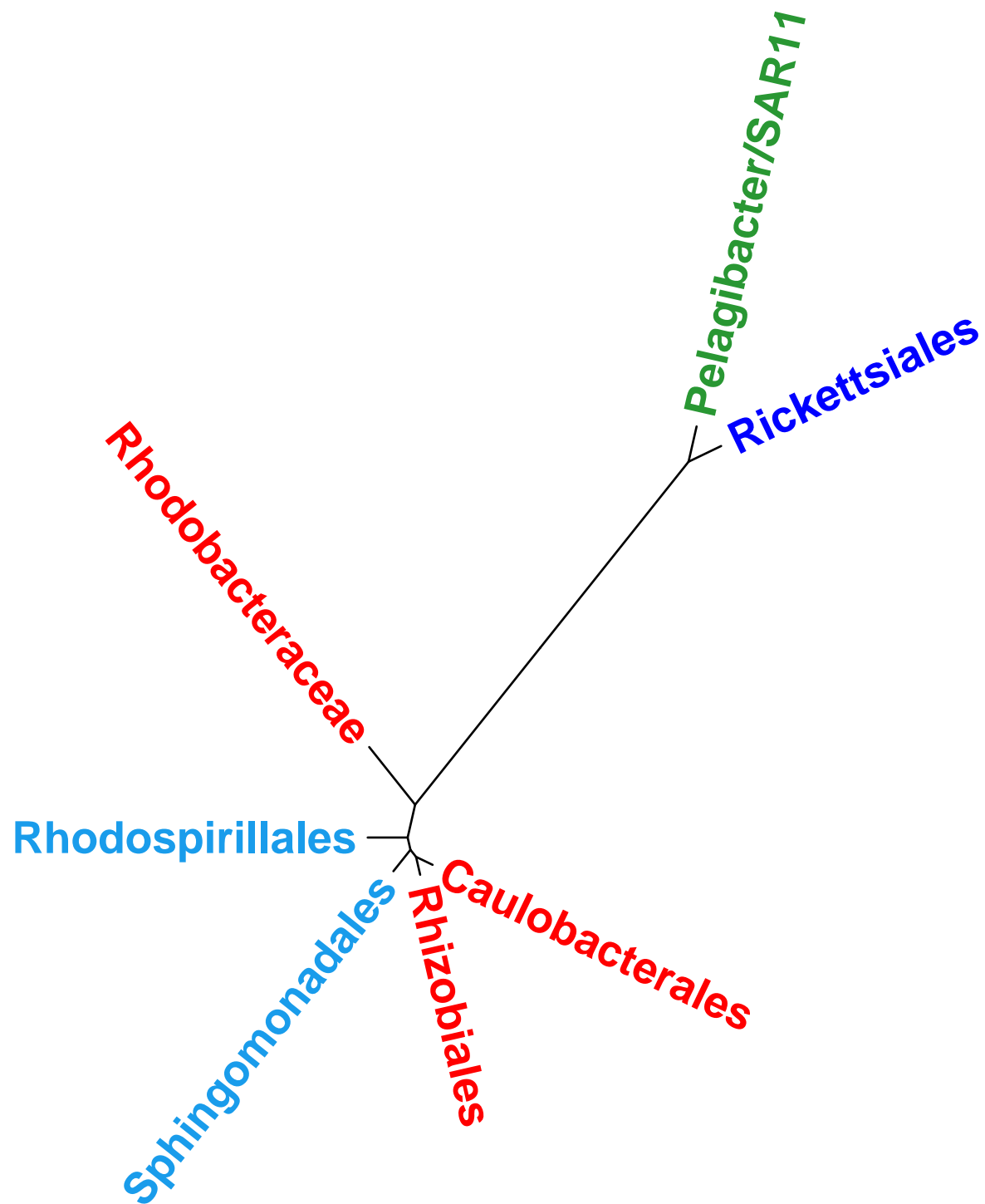

0.02

Supplement: Dataset S6 — Source code and data to reproduce Figure 7 . (ZIP) [file pcbi.1003454.s006.zip › Source_data_and_methods_for_Figure_7/alpha_tRNA_composition.neighbor.fig.pdf]

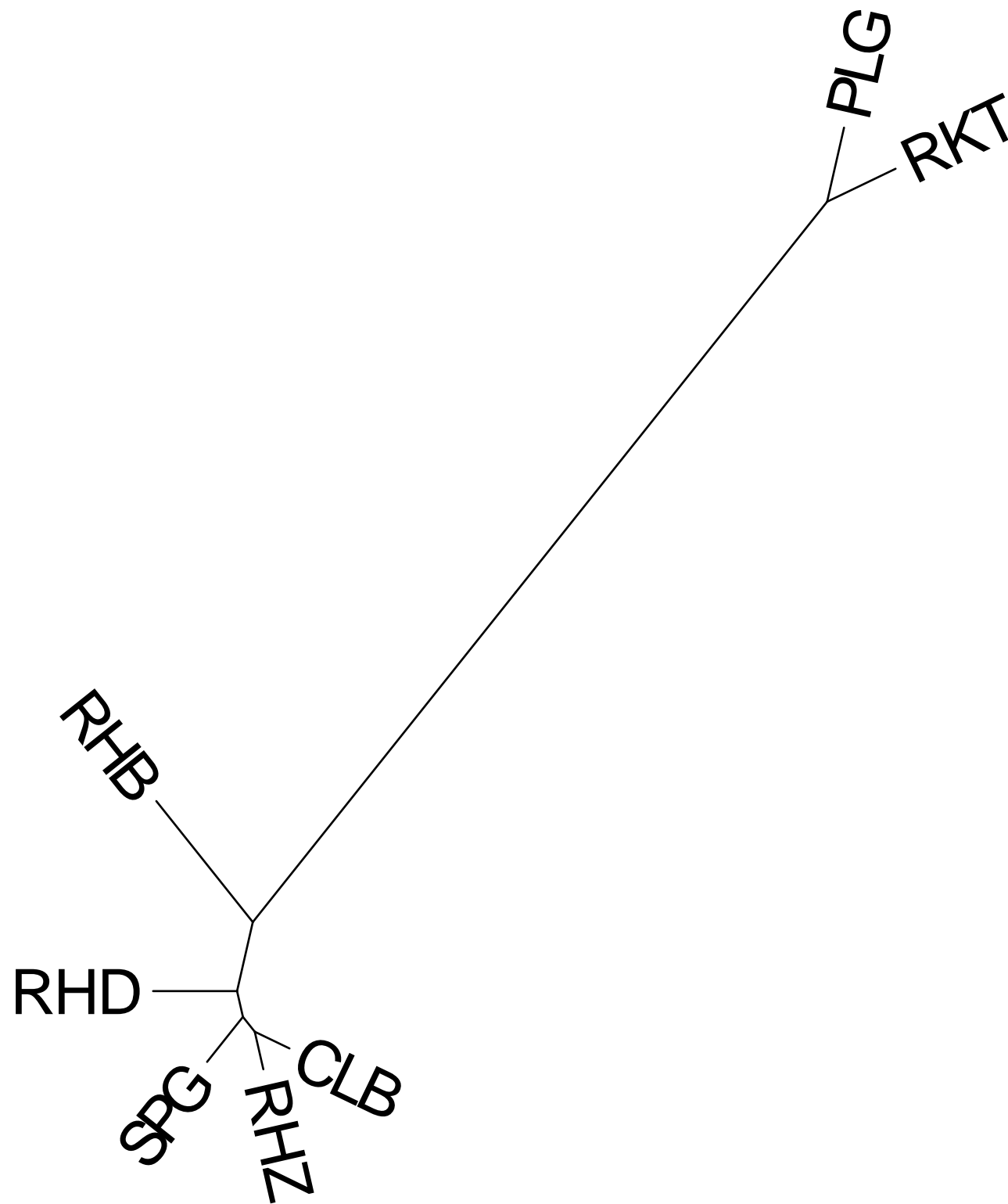

0.02

Supplement: Dataset S6 — Source code and data to reproduce Figure 7 . (ZIP) [file pcbi.1003454.s006.zip › Source_data_and_methods_for_Figure_7/alpha_tRNA_composition.neighbor.pdf]

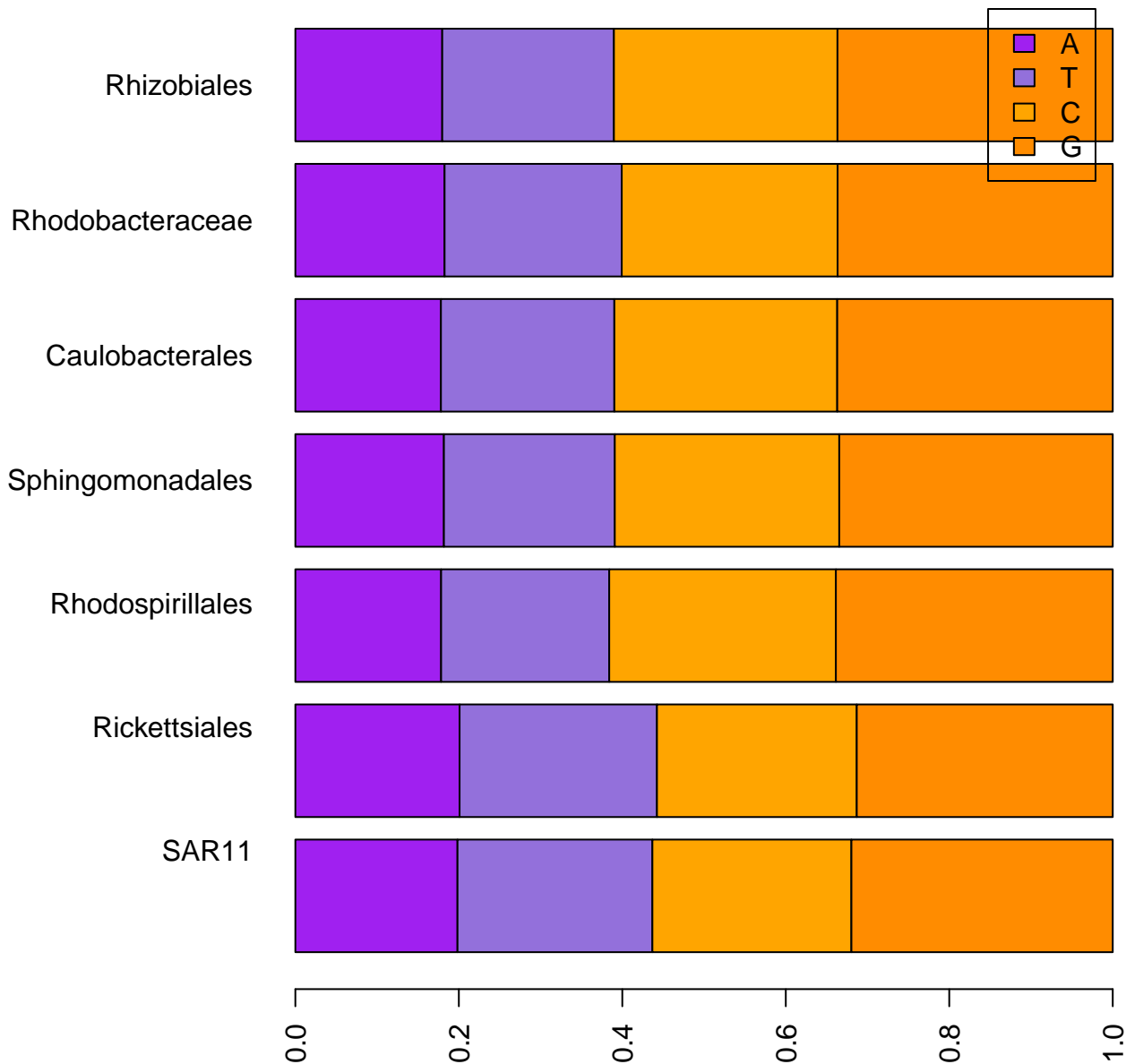

Supplement: Dataset S6 — Source code and data to reproduce Figure 7 . (ZIP) [file pcbi.1003454.s006.zip › Source_data_and_methods_for_Figure_7/Rplots.pdf]

## Frequency plots of residue in active site in HisRS

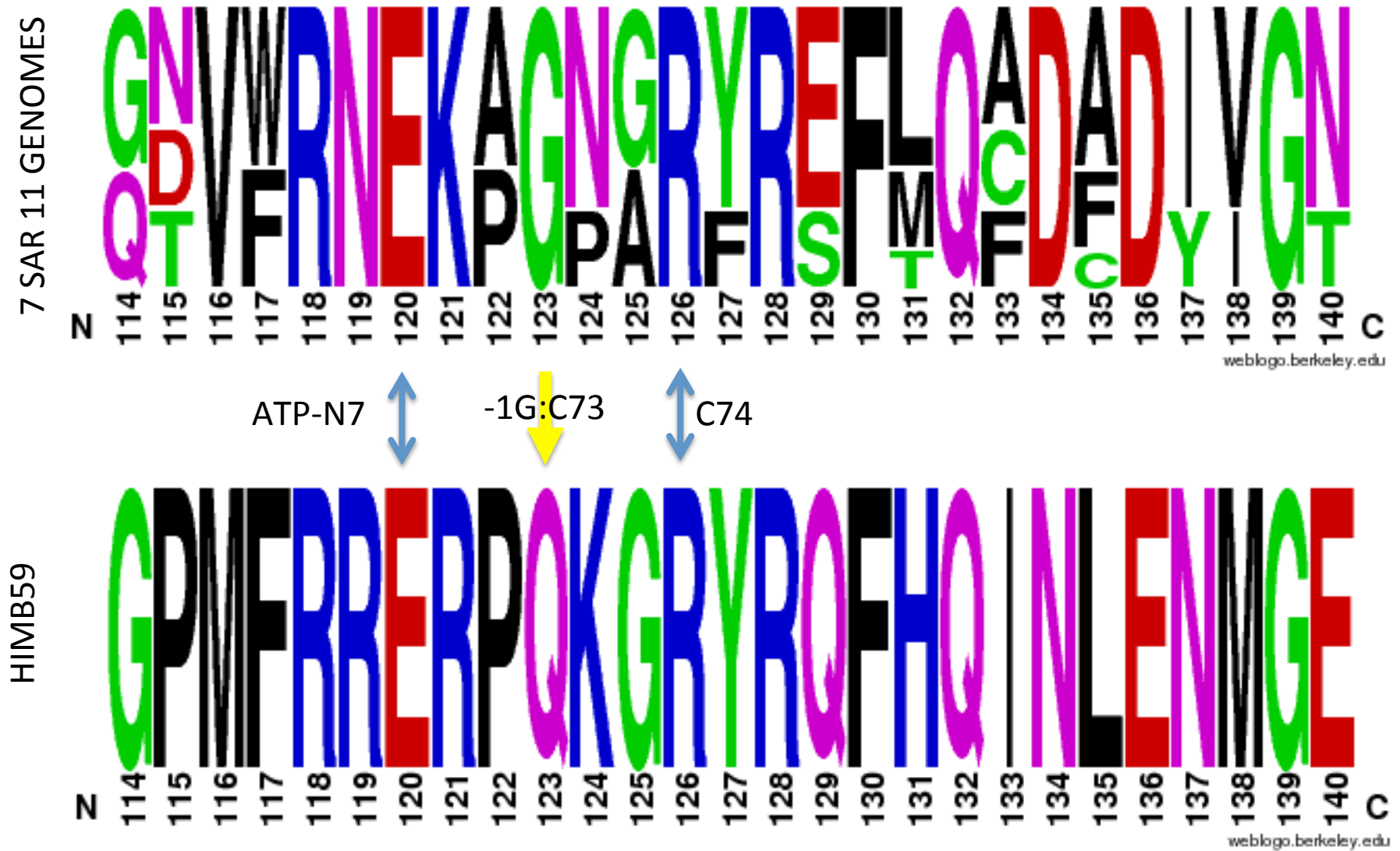

Supplement: Dataset S7 — Source code and data to reproduce Figure S1. (ZIP) [file pcbi.1003454.s007.zip › Source_data_and_methods_for_Supplementary_Figure_1/pel_hisRS_8genome.pdf]

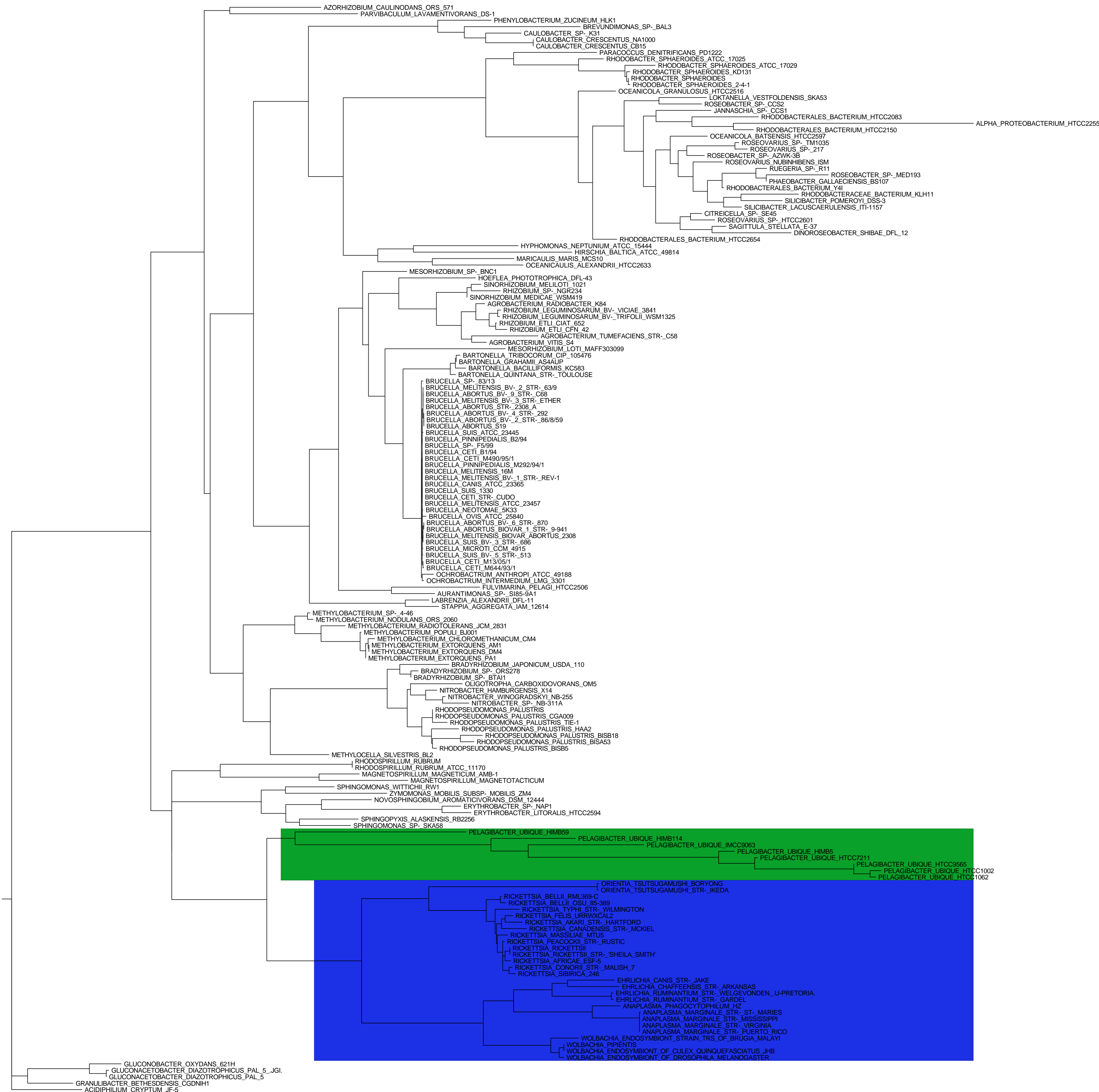

Supplement: Dataset S8 — Source code and data to reproduce Figures S3 and S4. (ZIP) [file pcbi.1003454.s008.zip › Source_data_and_methods_for_Supplementary_Figure_S3_and_S4/alpha_raxml.pdf]

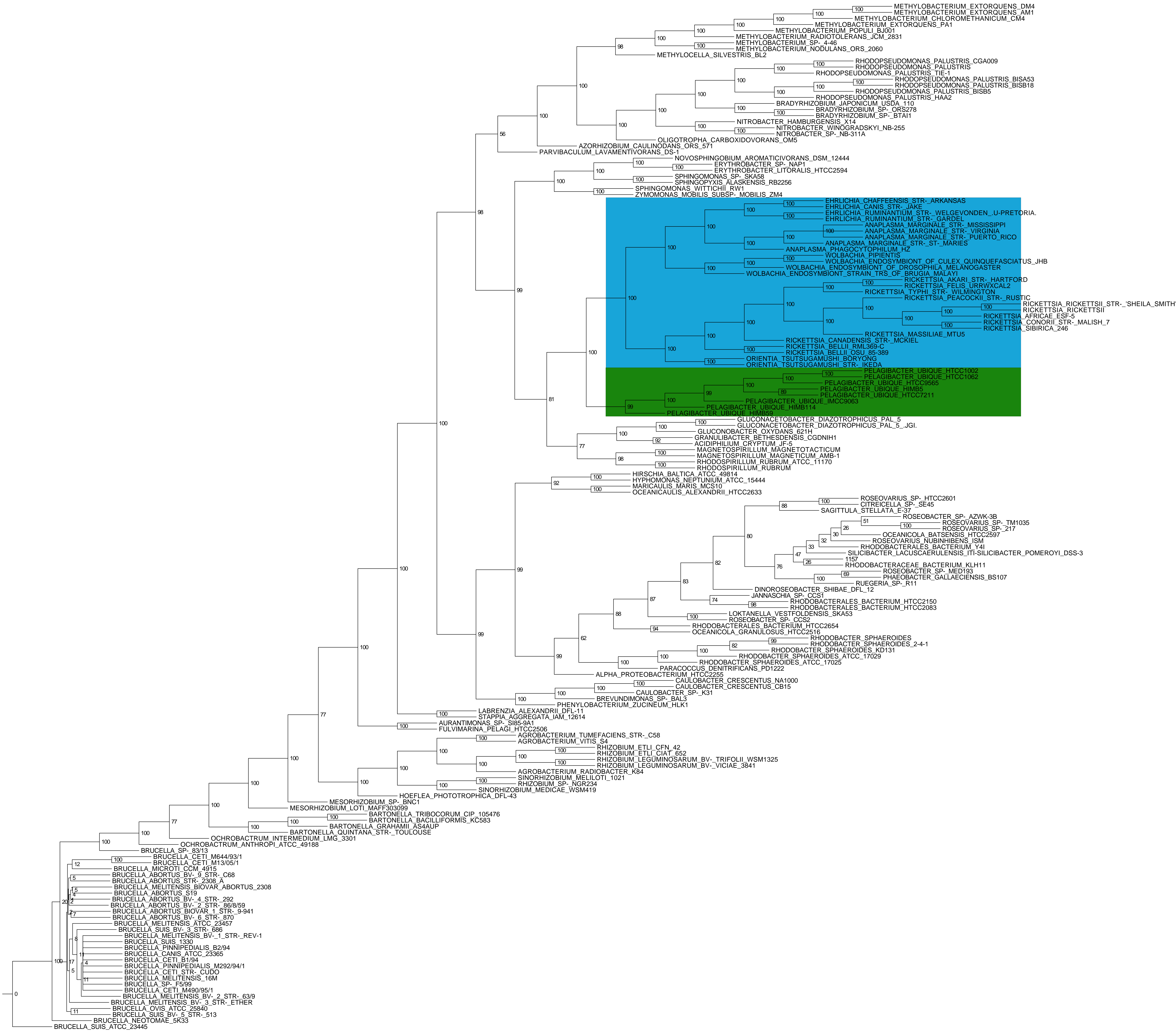

Supplement: Dataset S8 — Source code and data to reproduce Figures S3 and S4. (ZIP) [file pcbi.1003454.s008.zip › Source_data_and_methods_for_Supplementary_Figure_S3_and_S4/fasttree_boostrap_randomiso.pdf]
