# Supplementary material for: tRNA Signatures Reveal a Polyphyletic Origin of SAR11 Strains among Alphaproteobacteria
Source: PLoS Comput Biol. 2014 Feb 27;10(2):e1003454. doi: 10.1371/journal.pcbi.1003454 (PMC3937112; doi:10.1371/journal.pcbi.1003454)
Supplement: Table S1 — Numbers and base compositions of 214 alphaproteobacterial tRNA genes. This PDF file has its generating source file and raw data in CSV format attached. (PDF) [file pcbi.1003454.s013.pdf]

Supplementary Table S1 of Amrine *et al.* (2014)

Katherine C.H. Amrine, Wesley D. Swingley, and David H. Ardell

22 August 2013

This document presents Supplementary Table S1 of Amrine et al. (2014) "tRNA signatures reveal polyphyletic origins of streamlined SAR11 genomes among the Alphaproteobacteria" published in *PLoS Computational Biology.*

1 PDF attachments and how to regenerate this PDF file

The original LaTeX file that generated this PDF is attached to this PDF. To generate the PDF from the LaTeX attachment, extract the attachment and run `pdflatex` on it. The data table presented below is also attached to this PDF in comma-separated-values (CSV) format, to greater precision than presented here. Attachments may be added or extracted in Adobe Acrobat Pro or the open-source pdftk toolkit.

2 Description

The table presents data on numbers of tRNA genes and their base compositions among the 214 alphaproteobacterial genomes we analyzed.

Genome	#tRNAs	%A	%T	%C	%G	%G+C
WOLBACHIA SP- WRI	33	20.5	23.8	24.6	31.1	55.7
WOLBACHIA PIPIENTIS	34	20.5	23.8	24.6	31.0	55.6
WOLBACHIA ENDOSYMBIONT STRAIN TRS OF BRUGIA MALAYI	34	20.8	24.3	24.1	30.7	54.8
WOLBACHIA ENDOSYMBIONT OF MUSCIDIFURAX UNIRAPTOR	23	20.7	24.0	24.3	30.9	55.3
WOLBACHIA ENDOSYMBIONT OF DROSOPHILA SIMULANS	18	20.3	23.7	25.0	31.1	56.0
WOLBACHIA ENDOSYMBIONT OF DROSOPHILA MELANOGASTER	34	20.5	23.9	24.6	31.1	55.7
WOLBACHIA ENDOSYMBIONT OF DROSOPHILA ANANASSAE	28	19.9	24.1	24.4	31.7	56.1
WOLBACHIA ENDOSYMBIONT OF CULEX QUINQUEFASCIATUS JHB	34	20.5	23.8	24.6	31.0	55.6
RICKETTSIA TYPHI STR- WILMINGTON	33	20.4	24.3	24.4	30.8	55.3
RICKETTSIA SIBIRICA 246	33	19.9	24.1	24.6	31.4	56.0
RICKETTSIA RICKETTSII STR- IOWA	33	19.8	24.0	24.6	31.6	56.2
RICKETTSIA RICKETTSII STR- .SHEILA SMITH.	34	19.8	24.0	24.7	31.5	56.2
RICKETTSIA RICKETTSII	34	19.8	24.0	24.7	31.5	56.2
RICKETTSIA PROWAZEKII	32	20.2	24.1	24.5	31.2	55.7
RICKETTSIA PEACOCKII STR- RUSTIC	33	19.8	24.0	24.7	31.5	56.2
RICKETTSIA MASSILIAE MTU5	66	19.8	23.9	24.7	31.6	56.3
RICKETTSIA FELIS URRWXCAL2	33	19.9	24.0	24.6	31.5	56.1
RICKETTSIA ENDOSYMBIONT OF IXODES SCAPULARIS	32	20.1	24.0	24.6	31.3	56.0

Continued on next page

Genome	#tRNAs	%A	%T	%C	%G	%G+C
RICKETTSIA CONORII STR- MALISH 7	33	19.7	24.1	24.6	31.7	56.2
RICKETTSIA CANADENSIS STR- MCKIEL	66	20.2	24.2	24.4	31.1	55.5
RICKETTSIA BELLII RML369-C	33	19.8	24.3	24.4	31.5	55.9
RICKETTSIA BELLII OSU 85-389	65	19.8	24.3	24.4	31.5	55.9
RICKETTSIA AKARI STR- HARTFORD	66	19.9	24.3	24.4	31.5	55.9
RICKETTSIA AFRICAE ESF-5	66	19.8	23.9	24.8	31.5	56.3
ORIENTIA TSUTSUGAMUSHI STR- IKEDA	34	21.2	25.1	23.5	30.2	53.7
ORIENTIA TSUTSUGAMUSHI BORYONG	34	21.3	25.1	23.6	30.1	53.7
NEORICKETTSIA SENNETSU STR- MIYAYAMA	33	20.0	24.8	23.7	31.5	55.1
NEORICKETTSIA RISTICII STR- ILLINOIS	33	20.1	24.7	23.8	31.5	55.2
EHRLICHIA RUMINANTIUM STR- WELGEVONDEN .U-PRETORIA.	36	21.4	25.2	23.6	29.8	53.4
EHRLICHIA RUMINANTIUM STR- WELGEVONDEN .CIRAD.	36	21.5	25.2	23.6	29.8	53.4
EHRLICHIA RUMINANTIUM STR- GARDEL	36	21.4	25.2	23.6	29.8	53.4
EHRLICHIA CHAFFEENSIS STR- SAPULPA	26	21.0	24.8	24.1	30.2	54.2
EHRLICHIA CHAFFEENSIS STR- ARKANSAS	37	20.9	24.7	24.1	30.3	54.4
EHRLICHIA CANIS STR- JAKE	74	20.6	24.8	24.0	30.6	54.6
ANAPLASMA PHAGOCYTOPHILUM HZ	37	19.3	23.6	24.6	32.5	57.1
ANAPLASMA MARGINALE STR- VIRGINIA	37	18.8	23.0	25.3	32.9	58.2
ANAPLASMA MARGINALE STR- ST- MARIES	37	18.7	23.0	25.3	32.9	58.2
ANAPLASMA MARGINALE STR- PUERTO RICO	37	18.8	23.0	25.3	32.9	58.2
ANAPLASMA MARGINALE STR- MISSISSIPPI	37	18.8	23.0	25.3	32.9	58.2
ANAPLASMA MARGINALE STR- FLORIDA	36	18.8	23.0	25.3	33.0	58.2
PELAGIBACTER UBIQUE HIMB59	35	18.6	22.9	25.5	33.0	58.5
RHODOSPIRILLUM RUBRUM ATCC 11170	55	17.6	20.7	27.7	34.0	61.7
RHODOSPIRILLUM RUBRUM	51	17.7	20.7	27.7	33.9	61.6
RHODOSPIRILLUM CENTENUM SW	51	18.2	20.4	27.8	33.6	61.4
MAGNETOSPIRILLUM MAGNETOTACTICUM	83	17.5	20.4	28.2	34.0	62.1
MAGNETOSPIRILLUM MAGNETICUM AMB-1	49	17.5	20.7	27.7	34.0	61.7
GRANULIBACTER BETHESDENSIS CGDNIH1	52	18.0	20.1	27.8	34.0	61.8
GLUCONOBACTER OXYDANS 621H	55	18.5	20.8	27.4	33.4	60.8
GLUCONACETOBACTER DIAZOTROPHICUS PAL 5 .JGI.	55	17.7	20.6	27.8	34.0	61.7
GLUCONACETOBACTER DIAZOTROPHICUS PAL 5	101	17.9	20.6	27.6	33.9	61.5
ACIDIPHILIUM CRYPTUM JF-5	92	17.8	20.7	27.6	33.9	61.5
ZYMOMONAS MOBILIS SUBSP- MOBILIS ZM4	52	18.4	20.8	27.6	33.2	60.8
ZYMOMONAS MOBILIS SUBSP- MOBILIS ATCC 10988	46	18.5	20.9	27.5	33.1	60.6
SPHINGOPYXIS ALASKENSIS RB2256	90	18.1	20.6	27.7	33.6	61.3
SPHINGOMONAS WITTICHII RW1	96	17.9	20.5	27.7	33.9	61.6
SPHINGOMONAS SP- SKA58	58	18.4	20.8	27.6	33.2	60.9
NOVOSPHINGOBIUM AROMATICIVORANS DSM 12444	103	18.0	20.7	27.8	33.5	61.3
ERYTHROBACTER SP- SD-21	44	17.9	21.3	27.3	33.5	60.8
ERYTHROBACTER SP- NAP1	43	18.5	21.4	26.8	33.4	60.1
ERYTHROBACTER LITORALIS HTCC2594	86	18.3	21.6	26.8	33.3	60.1
PHENYLOBACTERIUM ZUCINEUM HLK1	43	17.6	20.6	27.6	34.1	61.8
CAULOBACTER SP- K31	97	17.7	21.2	27.3	33.7	61.0
CAULOBACTER CRESCENTUS NA1000	51	17.8	21.0	27.4	33.8	61.2
CAULOBACTER CRESCENTUS CB15	51	17.8	21.0	27.4	33.7	61.2

Continued on next page

Genome	#tRNAs	%A	%T	%C	%G	%G+C
BREVUNDIMONAS SP- BAL3	48	17.8	21.4	27.3	33.5	60.8
ASTICCACAULIS EXCENTRICUS CB 48	44	18.2	22.0	26.5	33.2	59.8
THALASSIOBIUM SP- R2A62	42	18.8	22.1	25.8	33.4	59.1
SULFITOBACTER SP- NAS-14-1	48	18.5	21.9	26.2	33.4	59.6
SULFITOBACTER SP- EE-36	49	18.6	21.9	26.3	33.2	59.5
SILICIBACTER SP- TRICHCH4B	59	18.9	21.7	26.1	33.3	59.4
SILICIBACTER SP- TM1040	98	18.5	21.8	26.1	33.5	59.7
SILICIBACTER POMEROYI DSS-3	52	18.1	21.9	26.2	33.7	60.0
SILICIBACTER LACUSCAERULENSIS ITI-1157	51	18.3	21.8	26.3	33.6	59.9
SAGITTULA STELLATA E-37	46	18.1	22.0	26.2	33.7	60.0
RUEGERIA SP- R11	58	18.4	21.8	26.1	33.6	59.8
ROSEOVARIUS SP- TM1035	48	18.5	21.4	26.6	33.5	60.1
ROSEOVARIUS SP- HTCC2601	55	18.5	21.4	26.5	33.5	60.1
ROSEOVARIUS SP- 217	43	18.2	21.5	26.5	33.8	60.3
ROSEOVARIUS NUBINHIBENS ISM	46	18.4	22.1	26.0	33.5	59.4
ROSEOBACTER SP- SK209-2-6	58	18.4	21.6	26.3	33.6	60.0
ROSEOBACTER SP- MED193	52	18.5	21.6	26.5	33.4	59.9
ROSEOBACTER SP- GAI101	45	18.3	22.2	25.8	33.6	59.5
ROSEOBACTER SP- CCS2	39	18.6	22.0	26.1	33.4	59.5
ROSEOBACTER SP- AZWK-3B	44	18.1	21.8	26.2	33.8	60.0
ROSEOBACTER LITORALIS OCH 149	38	18.4	22.1	25.9	33.6	59.5
ROSEOBACTER DENITRIFICANS OCH 114	38	18.2	22.0	26.0	33.7	59.7
RHODOBACTERALES BACTERIUM Y4I	57	18.2	22.0	26.0	33.8	59.8
RHODOBACTERALES BACTERIUM HTCC2654	43	17.8	21.5	26.5	34.2	60.7
RHODOBACTERALES BACTERIUM HTCC2150	39	18.8	21.9	25.9	33.4	59.3
RHODOBACTERALES BACTERIUM HTCC2083	40	18.1	22.2	26.1	33.6	59.6
RHODOBACTERACEAE BACTERIUM KLH11	51	18.2	21.9	26.3	33.6	59.9
RHODOBACTER SPHAEROIDES KD131	55	17.6	20.8	27.4	34.1	61.6
RHODOBACTER SPHAEROIDES ATCC 17029	111	17.7	20.9	27.4	34.0	61.4
RHODOBACTER SPHAEROIDES ATCC 17025	93	17.5	20.6	27.7	34.2	62.0
RHODOBACTER SPHAEROIDES 2-4-1	52	17.6	20.8	27.4	34.2	61.6
RHODOBACTER SPHAEROIDES	48	17.5	20.8	27.5	34.2	61.7
PHAEOBACTER GALLAECIENSIS BS107	61	18.5	21.7	26.2	33.6	59.8
PHAEOBACTER GALLAECIENSIS 2-10	57	18.4	21.8	26.2	33.6	59.8
PARACOCCUS DENITRIFICANS PD1222	99	17.9	21.1	27.3	33.7	61.0
OCTADECABACTER ANTARCTICUS 307	43	18.8	22.3	25.8	33.0	58.9
OCTADECABACTER ANTARCTICUS 238	42	18.5	22.3	25.8	33.3	59.1
OCEANICOLA GRANULOSUS HTCC2516	52	18.5	21.5	26.3	33.7	60.0
OCEANICOLA BATSENSIS HTCC2597	43	18.0	21.8	26.3	33.9	60.2
OCEANIBULBUS INDOLIFEX HEL-45	46	18.3	21.9	26.1	33.7	59.8
LOKTANELLA VESTFOLDENSIS SKA53	42	18.9	22.1	25.8	33.2	59.0
JANNASCHIA SP- CCS1	84	17.9	22.0	26.2	33.9	60.1
DINOROSEOBACTER SHIBAE DFL 12	86	18.1	21.8	26.3	33.7	60.1
CITREICELLA SP- SE45	56	18.5	21.6	26.4	33.5	59.9
ALPHA PROTEOBACTERIUM HTCC2255	71	19.0	22.3	25.8	32.9	58.7
OCEANICAULIS ALEXANDRII HTCC2633	44	17.8	21.0	27.1	34.1	61.1

Continued on next page

Genome	#tRNAs	%A	%T	%C	%G	%G+C
MARICAULIS MARIS MCS10	88	17.7	21.1	27.0	34.3	61.3
HYPHOMONAS NEPTUNIUM ATCC 15444	42	18.8	21.5	26.4	33.3	59.7
HIRSCHIA BALTICA ATCC 49814	38	18.3	22.2	26.1	33.4	59.5
LABRENZIA ALEXANDRII DFL-11	51	17.9	21.1	27.0	34.0	61.0
STAPPIA AGGREGATA IAM 12614	59	18.2	21.0	27.1	33.7	60.9
PSEUDOVIBRIO SP- JE062	85	18.0	21.4	26.8	33.8	60.6
PELAGIBACTER UBIQUE HIMB5	32	19.8	23.5	24.4	32.4	56.8
PELAGIBACTER UBIQUE HTCC7211	32	20.0	23.5	24.3	32.2	56.5
PELAGIBACTER UBIQUE HIMB114	32	19.5	23.4	25.2	31.9	57.1
PELAGIBACTER UBIQUE IMCC9063	32	20.7	24.1	24.1	31.1	55.1
PELAGIBACTER UBIQUE HTCC1062	31	20.2	24.5	23.7	31.6	55.3
PELAGIBACTER UBIQUE HTCC1002	31	20.1	24.7	23.6	31.6	55.2
PELAGIBACTER UBIQUE HTCC9565	30	20.0	24.3	23.9	31.8	55.7
XANTHOBACTER AUTOTROPHICUS PY2	92	17.9	20.3	27.8	34.0	61.8
SINORHIZOBIUM MELILOTI 1021	53	17.8	20.8	27.6	33.7	61.3
SINORHIZOBIUM MEDICAE WSM419	102	18.0	20.9	27.6	33.6	61.2
RHODOPSEUDOMONAS PALUSTRIS TIE-1	48	17.7	20.5	27.8	34.0	61.8
RHODOPSEUDOMONAS PALUSTRIS HAA2	96	17.6	20.7	27.8	34.0	61.8
RHODOPSEUDOMONAS PALUSTRIS CGA009	48	17.6	21.0	27.3	34.1	61.4
RHODOPSEUDOMONAS PALUSTRIS BISB5	94	17.5	20.8	27.6	34.1	61.7
RHODOPSEUDOMONAS PALUSTRIS BISB18	96	17.8	21.2	27.2	33.8	61.0
RHODOPSEUDOMONAS PALUSTRIS BISA53	96	17.6	21.1	27.4	34.0	61.4
RHODOPSEUDOMONAS PALUSTRIS	46	17.6	20.9	27.4	34.1	61.5
RHIZOBIUM SP- NGR234	53	17.8	20.7	27.7	33.8	61.5
RHIZOBIUM LEGUMINOSARUM BV- VICIAE 3841	52	18.6	21.3	27.1	33.0	60.1
RHIZOBIUM LEGUMINOSARUM BV- TRIFOLII WSM2304	100	18.6	21.4	27.0	33.0	60.0
RHIZOBIUM LEGUMINOSARUM BV- TRIFOLII WSM1325	99	18.6	21.5	27.0	32.9	59.9
RHIZOBIUM ETLI KIM 5	36	18.2	21.6	27.1	33.2	60.2
RHIZOBIUM ETLI IE4771	30	17.8	21.2	27.1	33.8	60.9
RHIZOBIUM ETLI GR56	31	19.2	21.3	27.1	32.5	59.5
RHIZOBIUM ETLI CIAT 894	28	18.7	21.6	26.5	33.2	59.7
RHIZOBIUM ETLI CIAT 652	51	18.5	21.3	27.1	33.1	60.1
RHIZOBIUM ETLI CFN 42	51	18.5	21.4	27.0	33.1	60.1
RHIZOBIUM ETLI BRASIL 5	28	18.7	21.2	27.1	32.9	60.0
RHIZOBIUM ETLI 8C-3	40	18.6	21.7	26.7	33.0	59.7
PARVIBACULUM LAVAMENTIVORANS DS-1	90	17.8	21.4	26.9	33.9	60.8
OLIGOTROPHA CARBOXIDOVORANS OM5	94	17.8	21.1	27.2	33.9	61.2
OCHROBACTRUM INTERMEDIUM LMG 3301	55	18.1	20.8	27.7	33.5	61.1
OCHROBACTRUM ANTHROPI ATCC 49188	57	18.1	21.0	27.5	33.4	60.9
NITROBACTER WINOGRADSKYI NB-255	46	18.0	21.2	27.2	33.6	60.8
NITROBACTER SP- NB-311A	47	18.1	21.3	27.1	33.5	60.6
NITROBACTER HAMBURGENSIS X14	96	17.8	21.0	27.2	33.9	61.2
METHYLOCELLA SILVESTRIS BL2	88	17.8	20.8	27.5	33.8	61.4
METHYLOBACTERIUM SP- 4-46	118	17.4	20.1	28.1	34.4	62.5
METHYLOBACTERIUM RADIOTOLERANS JCM 2831	53	17.7	20.6	27.7	34.0	61.7
METHYLOBACTERIUM POPULI BJ001	107	17.8	20.4	27.9	33.9	61.8

Continued on next page

Genome	#tRNAs	%A	%T	%C	%G	%G+C
METHYLOBACTERIUM NODULANS ORS 2060	122	17.6	20.2	28.0	34.2	62.2
METHYLOBACTERIUM EXTORQUENS PA1	106	17.8	20.2	28.1	33.8	61.9
METHYLOBACTERIUM EXTORQUENS DM4	58	17.7	20.3	28.0	34.0	62.0
METHYLOBACTERIUM EXTORQUENS AM1	56	17.8	20.4	28.0	33.9	61.9
METHYLOBACTERIUM CHLOROMETHANICUM CM4	105	17.8	20.3	28.1	33.9	62.0
MESORHIZOBIUM SP- BNC1	94	17.7	20.9	27.4	34.0	61.4
MESORHIZOBIUM LOTI MAFF303099	50	17.9	21.5	27.0	33.5	60.6
HYPHOMICROBIUM DENITRIFICANS ATCC 51888	45	18.5	20.8	27.4	33.3	60.7
HOEFLEA PHOTOTROPHICA DFL-43	47	18.4	21.0	27.1	33.5	60.5
FULVIMARINA PELAGI HTCC2506	54	17.9	21.0	27.3	33.9	61.1
CANDIDATUS LIBERIBACTER ASIATICUS STR- PSY62	85	19.3	23.0	25.5	32.2	57.7
CANDIDATUS HODGKINIA CICADICOLA DSEM	10	20.2	23.4	25.2	31.2	56.4
BRUCELLA SUIS BV- 5 STR- 513	49	17.8	21.1	27.4	33.8	61.1
BRUCELLA SUIS BV- 3 STR- 686	49	17.8	21.1	27.3	33.8	61.1
BRUCELLA SUIS ATCC 23445	54	17.8	20.9	27.5	33.8	61.3
BRUCELLA SUIS 1330	55	17.8	20.9	27.6	33.8	61.3
BRUCELLA SP- F5.99	48	17.9	21.2	27.2	33.8	61.0
BRUCELLA SP- 83.13	49	17.8	21.2	27.4	33.5	60.9
BRUCELLA PINNIPEDIALIS M292.94.1	49	17.8	21.1	27.3	33.7	61.1
BRUCELLA PINNIPEDIALIS M163.99.10	44	17.9	21.1	27.2	33.7	60.9
BRUCELLA PINNIPEDIALIS B2.94	49	17.8	21.1	27.3	33.7	61.1
BRUCELLA OVIS ATCC 25840	53	17.9	20.8	27.6	33.6	61.2
BRUCELLA NEOTOMAE 5K33	48	17.9	21.1	27.2	33.8	61.0
BRUCELLA MICROTI CCM 4915	55	17.8	20.9	27.6	33.7	61.3
BRUCELLA MELITENSIS BV- 3 STR- ETHER	49	17.8	21.1	27.3	33.7	61.0
BRUCELLA MELITENSIS BV- 2 STR- 63.9	49	17.8	21.1	27.3	33.7	61.0
BRUCELLA MELITENSIS BV- 1 STR- REV-1	48	17.9	21.1	27.2	33.8	61.0
BRUCELLA MELITENSIS BIOVAR ABORTUS 2308	55	17.8	20.9	27.5	33.8	61.3
BRUCELLA MELITENSIS ATCC 23457	55	17.8	20.9	27.5	33.7	61.3
BRUCELLA MELITENSIS 16M	54	17.9	20.9	27.5	33.8	61.3
BRUCELLA CETI STR- CUDO	50	17.9	20.9	27.5	33.7	61.1
BRUCELLA CETI M644.93.1	49	17.8	21.1	27.3	33.7	61.0
BRUCELLA CETI M490.95.1	48	17.9	21.1	27.2	33.8	61.0
BRUCELLA CETI M13.05.1	49	17.8	21.1	27.3	33.7	61.0
BRUCELLA CETI B1.94	48	17.9	21.1	27.2	33.8	61.0
BRUCELLA CANIS ATCC 23365	55	17.8	20.9	27.6	33.8	61.3
BRUCELLA ABORTUS STR- 2308 A	51	17.9	20.9	27.6	33.7	61.3
BRUCELLA ABORTUS S19	55	17.8	20.9	27.5	33.8	61.3
BRUCELLA ABORTUS BV- 9 STR- C68	49	17.8	21.1	27.3	33.8	61.1
BRUCELLA ABORTUS BV- 6 STR- 870	49	17.8	21.1	27.3	33.8	61.1
BRUCELLA ABORTUS BV- 4 STR- 292	49	17.8	21.1	27.3	33.8	61.1
BRUCELLA ABORTUS BV- 3 STR- TULYA	47	17.8	21.2	27.1	33.9	61.0
BRUCELLA ABORTUS BV- 2 STR- 86.8.59	49	17.8	21.1	27.3	33.8	61.1
BRUCELLA ABORTUS BIOVAR 1 STR- 9-941	55	17.8	20.9	27.5	33.8	61.3
BRADYRHIZOBIUM SP- ORS278	48	17.6	21.1	27.2	34.0	61.3
BRADYRHIZOBIUM SP- BTAI1	94	17.6	21.1	27.3	34.0	61.3

Continued on next page

Genome	#tRNAs	%A	%T	%C	%G	%G+C
BRADYRHIZOBIUM JAPONICUM USDA 110	49	17.5	21.1	27.6	33.9	61.4
BEIJERINCKIA INDICA SUBSP- INDICA ATCC 9039	51	17.9	21.1	27.2	33.9	61.1
BARTONELLA TRIBOCORUM CIP 105476	42	18.7	21.5	26.6	33.2	59.8
BARTONELLA QUINTANA STR- TOULOUSE	42	18.6	21.5	26.6	33.3	59.9
BARTONELLA HENSELAE	43	18.7	21.5	26.7	33.1	59.8
BARTONELLA GRAHAMII AS4AUP	42	18.7	21.5	26.6	33.2	59.8
BARTONELLA BACILLIFORMIS KC583	85	18.6	21.6	26.6	33.2	59.7
AZORHIZOBIUM CAULINODANS ORS 571	52	17.6	20.4	27.7	34.3	62.0
AURANTIMONAS SP- SI85-9A1	50	17.9	20.3	27.8	34.0	61.8
AGROBACTERIUM VITIS S4	54	18.8	21.3	27.0	33.0	59.9
AGROBACTERIUM TUMEFACIENS STR- C58	105	18.5	21.2	27.0	33.3	60.2
AGROBACTERIUM RADIOBACTER K84	51	18.8	21.6	26.7	32.9	59.6
