# Supplementary material for: tRNA Signatures Reveal a Polyphyletic Origin of SAR11 Strains among Alphaproteobacteria
Source: PLoS Comput Biol. 2014 Feb 27;10(2):e1003454. doi: 10.1371/journal.pcbi.1003454 (PMC3937112; doi:10.1371/journal.pcbi.1003454)
Supplement: Table S2 — Frequencies out of 100 bootstrap replicates that specific alphaproteobacterial test genomes classified into one among seven alphaproteobacterial clades. This PDF file has its generating source file and raw data in CSV format attached. (PDF) [file pcbi.1003454.s014.pdf]

Supplementary Table S2 of Amrine *et al.* (2014)

Katherine C.H. Amrine, Wesley D. Swingley, and David H. Ardell

07 January 2014

This document presents Supplementary Table S2 of Amrine et al. (2014) "tRNA signatures reveal polyphyletic origins of streamlined SAR11 genomes among the Alphaproteobacteria" published in *PLoS Computational Biology.*

1 PDF attachments and how to regenerate this PDF file

The original org-mode file that generated this PDF is attached to this PDF. To generate the PDF from the org-mode document attachment, extract the attachment, open it in an Emacs with org-mode and export the org-mode document to PDF with *e.g.* `^C ^E d`. The data table presented below is also attached to this PDF in comma-separated-values (CSV) format. Attachments may be added or extracted in Adobe Acrobat Pro or the open-source pdftk toolkit.

2 Description

The table presents the number of times out of 100 bootstrap replicates that specific alphaproteobacterial test genomes classified into one among seven alphaproteobacterial clades.

Genome	CIFs[1]	C[2]	H[3]	B[4]	Z[5]	S[6]	D[7]	K[8]
LABRENZIA ALEXANDRII DFL-11	all	0	0	1	53	37	9	0
LABRENZIA ALEXANDRII DFL-11	>0.5[9]	0	1	0	43	28	28	0
STAPPIA AGGREGATA IAM 12614	all	0	0	1	48	23	26	2
STAPPIA AGGREGATA IAM 12614	>0.5	0	4	0	45	14	36	1
PSEUDOVIBRIO SP- JE062	all	0	0	37	61	2	0	0
PSEUDOVIBRIO SP- JE062	>0.5	0	0	29	57	12	2	0
PELAGIBACTER UBIQUE HIMB5	all	13	3	3	48	9	6	18
PELAGIBACTER UBIQUE HIMB5	>0.5	0	13	3	59	5	12	8
PELAGIBACTER UBIQUE HTCC7211	all	7	2	2	54	13	9	13
PELAGIBACTER UBIQUE HTCC7211	>0.5	0	15	5	56	3	10	11
PELAGIBACTER UBIQUE HIMB114	all	1	0	0	70	3	11	15
PELAGIBACTER UBIQUE HIMB114	>0.5	0	9	1	66	2	12	10
PELAGIBACTER UBIQUE IMCC9063	all	2	1	3	80	12	2	0
PELAGIBACTER UBIQUE IMCC9063	>0.5	0	9	1	79	11	0	0
PELAGIBACTER UBIQUE HTCC1062	all	2	0	1	85	4	3	5
PELAGIBACTER UBIQUE HTCC1062	>0.5	0	9	3	80	2	5	1
PELAGIBACTER UBIQUE HTCC1002	all	4	1	2	81	4	3	5
PELAGIBACTER UBIQUE HTCC1002	>0.5	0	9	3	80	2	5	1
PELAGIBACTER UBIQUE HTCC9565	all	0	2	1	86	4	5	2
PELAGIBACTER UBIQUE HTCC9565	>0.5	0	9	3	79	4	5	0
PELAGIBACTER UBIQUE HIMB59	all	0	0	0	1	4	87	8
PELAGIBACTER UBIQUE HIMB59	>0.5	0	0	0	16	3	75	6

[1] Which CIFs were bootstrapped.
[2] Caulobacterales
[3] Hyphomonadaceae
[4] Rhodobacteraceae
[5] Rhizobiales
[6] Sphingomonadales
[7] Rhodospirillales
[8] Rickettsiales
[9] Only CIFs with Gorodkin heights > 0.5 bits were retained and bootstrapped.
