# Supplementary material for: tRNA Signatures Reveal a Polyphyletic Origin of SAR11 Strains among Alphaproteobacteria
Source: PLoS Comput Biol. 2014 Feb 27;10(2):e1003454. doi: 10.1371/journal.pcbi.1003454 (PMC3937112; doi:10.1371/journal.pcbi.1003454)
Supplement: Table S3 — Classifications of 214 alphaproteobacterial genomes across seven alphaproteobacterial clades after deletion of one of 22 different tRNA functional classes using the MLP multiway classifier model in WEKA. Genomes are ordered to match, top-to-bottom and left-to-right, Figure 5. Clades are symbolized as follows: K, Rickettsiales; D, Rhodospirillales; S, Sphingomonadales; C, Caulobacterales; B, Rhodobacteraceae; H, Hyphomonadaceae; Z, Rhizobiales. For each genome, the 22 clade classfications/functional class deletions are ordered by decreasing robustness of classifications to deletion over all genomes considered known (all but SAR11, Stappia, Labrenzia and Pseudovibrio). The class order is as follows: F,T,K,E,L,X,P (203 out of 203 genomes), S (202 genomes), A,I (201 genomes), N,Y,Q,M,J,W (200 genomes) V,D (199 genomes), C,H,R,G (197 genomes). This PDF file has its generating source file and raw data in CSV format attached. (PDF) [file pcbi.1003454.s015.pdf]

Supplementary Table S3 of Amrine *et al.* (2014)

Katherine C.H. Amrine, Wesley D. Swingley, and David H. Ardell

07 January 2014

This document presents Supplementary Table S3 of Amrine et al. (2014) "tRNA signatures reveal polyphyletic origins of streamlined SAR11 genomes among the Alphaproteobacteria" published in *PLoS Computational Biology.*

1 PDF attachments and how to regenerate this PDF file

The original org-mode file that generated this PDF is attached to this PDF. To generate the PDF from the org-mode document attachment, extract the attachment, open it in an Emacs with org-mode and export the org-mode document to PDF with *e.g.* `^C ^E d`. The data table presented below is also attached to this PDF in comma-separated-values (CSV) format. Attachments may be added or extracted in Adobe Acrobat Pro or the open-source pdftk toolkit.

2 Description

The table presents data on how the deletion of one of 22 different tRNA functional classes affects the tRNA CIF-based multiway phyloclassifications of 214 alphaproteobacterial genomes across seven alphaproteobacterial clades using the MLP multiway classifier model in WEKA. Here genomes are ordered to match, top-to-bottom and left-to-right, Figure 5 in Amrine et al. (2013). Clades are symbolized as follows: K => Rickettsiales, D => Rhodospirillales, S => Sphingomonadales, C => Caulobacterales, B => Rhodobacteraceae, H => Hyphomonadaceae, Z => Rhizobiales. For each genome, the 22 clade classifications / functional class deletions are ordered by decreasing robustness of classifications to deletion over all genomes considered "known" (all but SAR11, Stappia, Labrenzia and Pseudovibrio).

The class order is as follows: F,T,K,E,L,X,P (203 out of 203 genomes), S (202 genomes), A,I (201 genomes), N,Y,Q,M,J,W (200 genomes) V,D (199 genomes), C,H,R,G (197 genomes).

Genome	Classifications[1]
WOLBACHIA SP- WRI	KKKKKKKKKKKKKKKKKKKKKK
WOLBACHIA PIPIENTIS	KKKKKKKKKKKKKKKKKKKKKK
WOLBACHIA ENDOSYMBIONT STRAIN TRS OF BRUGIA MALAYI	KKKKKKKKKKKKKKKKKKKKKK
WOLBACHIA ENDOSYMBIONT OF MUSCIDIFURAX UNIRAPTOR	KKKKKKKKKKKKKKKKKKKKKK
WOLBACHIA ENDOSYMBIONT OF DROSOPHILA SIMULANS	KKKKKKKKKKKKKKKKKKKKKK
WOLBACHIA ENDOSYMBIONT OF DROSOPHILA MELANOGASTER	KKKKKKKKKKKKKKKKKKKKKK
WOLBACHIA ENDOSYMBIONT OF DROSOPHILA ANANASSAE	KKKKKKKKKKKKKKKKKKKKKK
WOLBACHIA ENDOSYMBIONT OF CULEX QUINQUEFASCIATUS JHB	KKKKKKKKKKKKKKKKKKKKKK
RICKETTSIA TYPHI STR- WILMINGTON	KKKKKKKKKKKKKKKKKKKKKK
RICKETTSIA SIBIRICA 246	KKKKKKKKKKKKKKKKKKKKKK
RICKETTSIA RICKETTSII STR- IOWA	KKKKKKKKKKKKKKKKKKKKKK
RICKETTSIA RICKETTSII STR- ‘SHEILA SMITH’	KKKKKKKKKKKKKKKKKKKKKK
RICKETTSIA RICKETTSII	KKKKKKKKKKKKKKKKKKKKKK
RICKETTSIA PROWAZEKII	KKKKKKKKKKKKKKKKKKKKKK
RICKETTSIA PEACOCKII STR- RUSTIC	KKKKKKKKKKKKKKKKKKKKKK
RICKETTSIA MASSILIAE MTU5	KKKKKKKKKKKKKKKKKKKKKK
RICKETTSIA FELIS URRWXCAL2	KKKKKKKKKKKKKKKKKKKKKK
RICKETTSIA ENDOSYMBIONT OF IXODES SCAPULARIS	KKKKKKKKKKKKKKKKKKKKKK
RICKETTSIA CONORII STR- MALISH 7	KKKKKKKKKKKKKKKKKKKKKK
RICKETTSIA CANADENSIS STR- MCKIEL	KKKKKKKKKKKKKKKKKKKKKK
RICKETTSIA BELLII RML369-C	KKKKKKKKKKKKKKKKKKKKKK
RICKETTSIA BELLII OSU 85-389	KKKKKKKKKKKKKKKKKKKKKK
RICKETTSIA AKARI STR- HARTFORD	KKKKKKKKKKKKKKKKKKKKKK
RICKETTSIA AFRICAE ESF-5	KKKKKKKKKKKKKKKKKKKKKK
ORIENTIA TSUTSUGAMUSHI STR- IKEDA	KKKKKKKKKKKKKKKKKKKKKK
ORIENTIA TSUTSUGAMUSHI BORYONG	KKKKKKKKKKKKKKKKKKKKKK
NEORICKETTSIA SENNETSU STR- MIYAYAMA	KKKKKKKKKKKKKKKKKKKKKK
NEORICKETTSIA RISTICII STR- ILLINOIS	KKKKKKKKKKKKKKKKKKKKKK
EHRLICHIA RUMINANTIUM STR- WELGEVONDEN (U-PRETORIA)	KKKKKKKKKKKKKKKKKKKKKK
EHRLICHIA RUMINANTIUM STR- WELGEVONDEN (CIRAD)	KKKKKKKKKKKKKKKKKKKKKK
EHRLICHIA RUMINANTIUM STR- GARDEL	KKKKKKKKKKKKKKKKKKKKKK
EHRLICHIA CHAFFEENSIS STR- SAPULPA	KKKKKKKKKKKKKKKKKKKKKK
EHRLICHIA CHAFFEENSIS STR- ARKANSAS	KKKKKKKKKKKKKKKKKKKKKK
EHRLICHIA CANIS STR- JAKE	KKKKKKKKKKKKKKKKKKKKKK

Continued on next page

Genome	Classifications[1]
ANAPLASMA PHAGOCYTOPHILUM HZ	KKKKKKKKKKKKKKKKKKKKKK
ANAPLASMA MARGINALE STR- VIRGINIA	KKKKKKKKKKKKKKKKKKKKKK
ANAPLASMA MARGINALE STR- ST- MARIES	KKKKKKKKKKKKKKKKKKKKKK
ANAPLASMA MARGINALE STR- PUERTO RICO	KKKKKKKKKKKKKKKKKKKKKK
ANAPLASMA MARGINALE STR- MISSISSIPPI	KKKKKKKKKKKKKKKKKKKKKK
ANAPLASMA MARGINALE STR- FLORIDA	KKKKKKKKKKKKKKKKKKKKKK
PELAGIBACTER UBIQUE HIMB59	DDDDDDDDDDDDDDDDDDDDKD
RHODOSPIRILLUM RUBRUM ATCC 11170	DDDDDDDDDDDDDDDDDDDDDD
RHODOSPIRILLUM RUBRUM	DDDDDDDDDDDDDDDDDDDDDD
RHODOSPIRILLUM CENTENUM SW	DDDDDDDDDDDDDDDDDDDDDD
MAGNETOSPIRILLUM MAGNETOTACTICUM	DDDDDDDDDDDDDDDDDDDDDD
MAGNETOSPIRILLUM MAGNETICUM AMB-1	DDDDDDDDDDDDDDDDDDDDDD
GRANULIBACTER BETHESDENSIS CGDNIH1	DDDDDDDDDDDDDDDDDDDDDD
GLUCONOBACTER OXYDANS 621H	DDDDDDDDDDDDDDDDDDDDDD
GLUCONACETOBACTER DIAZOTROPHICUS PAL 5 (JGI)	DDDDDDDDDDDDDDDDDDDDDD
GLUCONACETOBACTER DIAZOTROPHICUS PAL 5	DDDDDDDDDDDDDDDDDDDDDD
ACIDIPHILIUM CRYPTUM JF-5	DDDDDDDDDDDDDDDDDDDDDD
ZYMOMONAS MOBILIS SUBSP- MOBILIS ZM4	SSSSSSSSSSSSSSSSSSSSSS
ZYMOMONAS MOBILIS SUBSP- MOBILIS ATCC 10988	SSSSSSSSSSSSSSSSSSSSSS
SPHINGOPYXIS ALASKENSIS RB2256	SSSSSSSSSSSSSSSSSSSSSS
SPHINGOMONAS WITTICHII RW1	SSSSSSSSSSSSSSSSSSSSSS
SPHINGOMONAS SP- SKA58	SSSSSSSSSSSSSSSSSSSSSS
NOVOSPHINGOBIUM AROMATICIVORANS DSM 12444	SSSSSSSSSSSSSSSSSSSSSS
ERYTHROBACTER SP- SD-21	SSSSSSSSSSSSSSSSSSSSSS
ERYTHROBACTER SP- NAP1	SSSSSSSSSSSSSSSSSSSSSS
ERYTHROBACTER LITORALIS HTCC2594	SSSSSSSSSSSSSSSSSSSSSS
PHENYLOBACTERIUM ZUCINEUM HLK1	CCCCCCCCCCCCCCCCCCZZZZ
CAULOBACTER SP- K31	CCCCCCCCCCCCCCCCCCZZZZ
CAULOBACTER CRESCENTUS NA1000	CCCCCCCCCCCCCCCCCCZZZZ
CAULOBACTER CRESCENTUS CB15	CCCCCCCCCCCCCCCCCCZZZZ
BREVUNDIMONAS SP- BAL3	CCCCCCCCCCCCCCCCCCZZZZ
ASTICCACAULIS EXCENTRICUS CB 48	CCCCCCCCCCCCCCCCCCZZZZ
THALASSIOBIUM SP- R2A62	BBBBBBBBBBBBBBBBBBBBBB
SULFITOBACTER SP- NAS-14-1	BBBBBBBBBBBBBBBBBBBBBB
SULFITOBACTER SP- EE-36	BBBBBBBBBBBBBBBBBBBBBB
SILICIBACTER SP- TRICHCH4B	BBBBBBBBBBBBBBBBBBBBBB
SILICIBACTER SP- TM1040	BBBBBBBBBBBBBBBBBBBBBB
SILICIBACTER POMEROYI DSS-3	BBBBBBBBBBBBBBBBBBBBBB

Continued on next page

Genome	Classifications[1]
SILICIBACTER LACUSCAERULENSIS ITI-1157	BBBBBBBBBBBBBBBBBBBBBB
SAGITTULA STELLATA E-37	BBBBBBBBBBBBBBBBBBBBBB
RUEGERIA SP- R11	BBBBBBBBBBBBBBBBBBBBBB
ROSEOVARIUS SP- TM1035	BBBBBBBBBBBBBBBBBBBBBB
ROSEOVARIUS SP- HTCC2601	BBBBBBBBBBBBBBBBBBBBBB
ROSEOVARIUS SP- 217	BBBBBBBBBBBBBBBBBBBBBB
ROSEOVARIUS NUBINHIBENS ISM	BBBBBBBBBBBBBBBBBBBBBB
ROSEOBACTER SP- SK209-2-6	BBBBBBBBBBBBBBBBBBBBBB
ROSEOBACTER SP- MED193	BBBBBBBBBBBBBBBBBBBBBB
ROSEOBACTER SP- GAI101	BBBBBBBBBBBBBBBBBBBBBB
ROSEOBACTER SP- CCS2	BBBBBBBBBBBBBBBBBBBBBB
ROSEOBACTER SP- AZWK-3B	BBBBBBBBBBBBBBBBBBBBBB
ROSEOBACTER LITORALIS OCH 149	BBBBBBBBBBBBBBBBBBBBBB
ROSEOBACTER DENITRIFICANS OCH 114	BBBBBBBBBBBBBBBBBBBBBB
RHODOBACTERALES BACTERIUM Y4I	BBBBBBBBBBBBBBBBBBBBBB
RHODOBACTERALES BACTERIUM HTCC2654	BBBBBBBBBBBBBBBBBBBBBB
RHODOBACTERALES BACTERIUM HTCC2150	BBBBBBBBBBBBBBBBBBBBBB
RHODOBACTERALES BACTERIUM HTCC2083	BBBBBBBBBBBBBBBBBBBBBB
RHODOBACTERACEAE BACTERIUM KLH11	BBBBBBBBBBBBBBBBBBBBBB
RHODOBACTER SPHAEROIDES KD131	BBBBBBBBBBBBBBBBBBBBBB
RHODOBACTER SPHAEROIDES ATCC 17029	BBBBBBBBBBBBBBBBBBBBBB
RHODOBACTER SPHAEROIDES ATCC 17025	BBBBBBBBBBBBBBBBBBBBBB
RHODOBACTER SPHAEROIDES 2-4-1	BBBBBBBBBBBBBBBBBBBBBB
RHODOBACTER SPHAEROIDES	BBBBBBBBBBBBBBBBBBBBBB
PHAEOBACTER GALLAECIENSIS BS107	BBBBBBBBBBBBBBBBBBBBBB
PHAEOBACTER GALLAECIENSIS 2-10	BBBBBBBBBBBBBBBBBBBBBB
PARACOCCUS DENITRIFICANS PD1222	BBBBBBBBBBBBBBBBBBBBBB
OCTADECABACTER ANTARCTICUS 307	BBBBBBBBBBBBBBBBBBBBBB
OCTADECABACTER ANTARCTICUS 238	BBBBBBBBBBBBBBBBBBBBBB
OCEANICOLA GRANULOSUS HTCC2516	BBBBBBBBBBBBBBBBBBBBBB
OCEANICOLA BATSENSIS HTCC2597	BBBBBBBBBBBBBBBBBBBBBB
OCEANIBULBUS INDOLIFEX HEL-45	BBBBBBBBBBBBBBBBBBBBBB
LOKTANELLA VESTFOLDENSIS SKA53	BBBBBBBBBBBBBBBBBBBBBB
JANNASCHIA SP- CCS1	BBBBBBBBBBBBBBBBBBBBBB
DINOROSEOBACTER SHIBAE DFL 12	BBBBBBBBBBBBBBBBBBBBBB
CITREICELLA SP- SE45	BBBBBBBBBBBBBBBBBBBBBB
ALPHA PROTEOBACTERIUM HTCC2255	BBBBBBBBBBBBBBBBBBBBBB
OCEANICAULIS ALEXANDRII HTCC2633	HHHHHHHHHHHZZHHHZZHHHH

Continued on next page

Genome	Classifications[1]
MARICAULIS MARIS MCS10	HHHHHHHHHHSHHSSSSSHHHH
HYPHOMONAS NEPTUNIUM ATCC 15444	HHHHHHHHCCCCCCCCCCHHHH
HIRSCHIA BALTICA ATCC 49814	HHHHHHHBBBBBBBBBBBHHHH
LABRENZIA ALEXANDRII DFL-11	SDSZDZZZSZZSSZZSZZZSDS
STAPPIA AGGREGATA IAM 12614	DDDZZZZZZDZDDDDDDDDDDZ
PSEUDOVIBRIO SP- JE062	ZZBBBBZZZZZBBZZZZZBBBB
PELAGIBACTER UBIQUE HIMB5	ZZKDZHHZZZZCZZZZZZZZZZ
PELAGIBACTER UBIQUE HTCC7211	ZZHDZZZZZZZBZZZZZZZZZZ
PELAGIBACTER UBIQUE HIMB114	ZZZZZZZZZZZZZZZZZZZZZZ
PELAGIBACTER UBIQUE IMCC9063	ZZZZZZZZZZZZZZZZZZZZZZ
PELAGIBACTER UBIQUE HTCC1062	ZZZZZZZZZZZZZZZZZZZZZZ
PELAGIBACTER UBIQUE HTCC1002	ZZZZZZZZZZZZZZZZZZZZZZ
PELAGIBACTER UBIQUE HTCC9565	ZZZZZZZZZZZZZZZZZZZZZZ
XANTHOBACTER AUTOTROPHICUS PY2	ZZZZZZZZZZZZZZZZZZZZZZ
SINORHIZOBIUM MELILOTI 1021	ZZZZZZZZZZZZZZZZZZZZZZ
SINORHIZOBIUM MEDICAE WSM419	ZZZZZZZZZZZZZZZZZZZZZZ
RHODOPSEUDOMONAS PALUSTRIS TIE-1	ZZZZZZZZZZZZZZZZZZZZZZ
RHODOPSEUDOMONAS PALUSTRIS HAA2	ZZZZZZZZZZZZZZZZZZZZZZ
RHODOPSEUDOMONAS PALUSTRIS CGA009	ZZZZZZZZZZZZZZZZZZZZZZ
RHODOPSEUDOMONAS PALUSTRIS BISB5	ZZZZZZZZZZZZZZZZZZZZZZ
RHODOPSEUDOMONAS PALUSTRIS BISB18	ZZZZZZZZZZZZZZZZZZZZZZ
RHODOPSEUDOMONAS PALUSTRIS BISA53	ZZZZZZZZZZZZZZZZZZZZZZ
RHODOPSEUDOMONAS PALUSTRIS	ZZZZZZZZZZZZZZZZZZZZZZ
RHIZOBIUM SP- NGR234	ZZZZZZZZZZZZZZZZZZZZZZ
RHIZOBIUM LEGUMINOSARUM BV- VICIAE 3841	ZZZZZZZZZZZZZZZZZZZZZZ
RHIZOBIUM LEGUMINOSARUM BV- TRIFOLII WSM2304	ZZZZZZZZZZZZZZZZZZZZZZ
RHIZOBIUM LEGUMINOSARUM BV- TRIFOLII WSM1325	ZZZZZZZZZZZZZZZZZZZZZZ
RHIZOBIUM ETLI KIM 5	ZZZZZZZZZZZZZZZZZZZZZZ
RHIZOBIUM ETLI IE4771	ZZZZZZZZZZZZZZZZZZZZZZ
RHIZOBIUM ETLI GR56	ZZZZZZZZZZZZZZZZZZZZZZ
RHIZOBIUM ETLI CIAT 894	ZZZZZZZZZZZZZZZZZZZZZZ
RHIZOBIUM ETLI CIAT 652	ZZZZZZZZZZZZZZZZZZZZZZ
RHIZOBIUM ETLI CFN 42	ZZZZZZZZZZZZZZZZZZZZZZ
RHIZOBIUM ETLI BRASIL 5	ZZZZZZZZZZZZZZZZZZZZZZ
RHIZOBIUM ETLI 8C-3	ZZZZZZZZZZZZZZZZZZZZZZ
PARVIBACULUM LAVAMENTIVORANS DS-1	ZZZZZZZZZZZZZZZZZZZZZZ
OLIGOTROPHA CARBOXIDOVORANS OM5	ZZZZZZZZZZZZZZZZZZZZZZ
OCHROBACTRUM INTERMEDIUM LMG 3301	ZZZZZZZZZZZZZZZZZZZZZZ

Continued on next page

Genome	Classifications[1]
OCHROBACTRUM ANTHROPI ATCC 49188	ZZZZZZZZZZZZZZZZZZZZZZ
NITROBACTER WINOGRADSKYI NB-255	ZZZZZZZZZZZZZZZZZZZZZZ
NITROBACTER SP- NB-311A	ZZZZZZZZZZZZZZZZZZZZZZ
NITROBACTER HAMBURGENSIS X14	ZZZZZZZZZZZZZZZZZZZZZZ
METHYLOCELLA SILVESTRIS BL2	ZZZZZZZZZZZZZZZZZZZZZZ
METHYLOBACTERIUM SP- 4-46	ZZZZZZZZZZZZZZZZZZZZZZ
METHYLOBACTERIUM RADIOTOLERANS JCM 2831	ZZZZZZZZZZZZZZZZZZZZZZ
METHYLOBACTERIUM POPULI BJ001	ZZZZZZZZZZZZZZZZZZZZZZ
METHYLOBACTERIUM NODULANS ORS 2060	ZZZZZZZZZZZZZZZZZZZZZZ
METHYLOBACTERIUM EXTORQUENS PA1	ZZZZZZZZZZZZZZZZZZZZZZ
METHYLOBACTERIUM EXTORQUENS DM4	ZZZZZZZZZZZZZZZZZZZZZZ
METHYLOBACTERIUM EXTORQUENS AM1	ZZZZZZZZZZZZZZZZZZZZZZ
METHYLOBACTERIUM CHLOROMETHANICUM CM4	ZZZZZZZZZZZZZZZZZZZZZZ
MESORHIZOBIUM SP- BNC1	ZZZZZZZZZZZZZZZZZZZZZZ
MESORHIZOBIUM LOTI MAFF303099	ZZZZZZZZZZZZZZZZZZZZZZ
HYPHOMICROBIUM DENITRIFICANS ATCC 51888	ZZZZZZZZZZZZZZZZZZZZZZ
HOEFLEA PHOTOTROPHICA DFL-43	ZZZZZZZZZZZZZZZZZZZZZZ
FULVIMARINA PELAGI HTCC2506	ZZZZZZZZZZZZZZZZZZZZZZ
CANDIDATUS LIBERIBACTER ASIATICUS STR- PSY62	ZZZZZZZZZZZZZZZZZZZZZZ
CANDIDATUS HODGKINIA CICADICOLA DSEM	ZZZZZZZZZZZZZZZZZZZZZZ
BRUCELLA SUIS BV- 5 STR- 513	ZZZZZZZZZZZZZZZZZZZZZZ
BRUCELLA SUIS BV- 3 STR- 686	ZZZZZZZZZZZZZZZZZZZZZZ
BRUCELLA SUIS ATCC 23445	ZZZZZZZZZZZZZZZZZZZZZZ
BRUCELLA SUIS 1330	ZZZZZZZZZZZZZZZZZZZZZZ
BRUCELLA SP- F5/99	ZZZZZZZZZZZZZZZZZZZZZZ
BRUCELLA SP- 83/13	ZZZZZZZZZZZZZZZZZZZZZZ
BRUCELLA PINNIPEDIALIS M292/94/1	ZZZZZZZZZZZZZZZZZZZZZZ
BRUCELLA PINNIPEDIALIS M163/99/10	ZZZZZZZZZZZZZZZZZZZZZZ
BRUCELLA PINNIPEDIALIS B2/94	ZZZZZZZZZZZZZZZZZZZZZZ
BRUCELLA OVIS ATCC 25840	ZZZZZZZZZZZZZZZZZZZZZZ
BRUCELLA NEOTOMAE 5K33	ZZZZZZZZZZZZZZZZZZZZZZ
BRUCELLA MICROTI CCM 4915	ZZZZZZZZZZZZZZZZZZZZZZ
BRUCELLA MELITENSIS BV- 3 STR- ETHER	ZZZZZZZZZZZZZZZZZZZZZZ
BRUCELLA MELITENSIS BV- 2 STR- 63/9	ZZZZZZZZZZZZZZZZZZZZZZ
BRUCELLA MELITENSIS BV- 1 STR- REV-1	ZZZZZZZZZZZZZZZZZZZZZZ
BRUCELLA MELITENSIS BIOVAR ABORTUS 2308	ZZZZZZZZZZZZZZZZZZZZZZ
BRUCELLA MELITENSIS ATCC 23457	ZZZZZZZZZZZZZZZZZZZZZZ
BRUCELLA MELITENSIS 16M	ZZZZZZZZZZZZZZZZZZZZZZ

Continued on next page

Genome	Classifications[1]
BRUCELLA CETI STR- CUDO	ZZZZZZZZZZZZZZZZZZZZZZ
BRUCELLA CETI M644/93/1	ZZZZZZZZZZZZZZZZZZZZZZ
BRUCELLA CETI M490/95/1	ZZZZZZZZZZZZZZZZZZZZZZ
BRUCELLA CETI M13/05/1	ZZZZZZZZZZZZZZZZZZZZZZ
BRUCELLA CETI B1/94	ZZZZZZZZZZZZZZZZZZZZZZ
BRUCELLA CANIS ATCC 23365	ZZZZZZZZZZZZZZZZZZZZZZ
BRUCELLA ABORTUS STR- 2308 A	ZZZZZZZZZZZZZZZZZZZZZZ
BRUCELLA ABORTUS S19	ZZZZZZZZZZZZZZZZZZZZZZ
BRUCELLA ABORTUS BV- 9 STR- C68	ZZZZZZZZZZZZZZZZZZZZZZ
BRUCELLA ABORTUS BV- 6 STR- 870	ZZZZZZZZZZZZZZZZZZZZZZ
BRUCELLA ABORTUS BV- 4 STR- 292	ZZZZZZZZZZZZZZZZZZZZZZ
BRUCELLA ABORTUS BV- 3 STR- TULYA	ZZZZZZZZZZZZZZZZZZZZZZ
BRUCELLA ABORTUS BV- 2 STR- 86/8/59	ZZZZZZZZZZZZZZZZZZZZZZ
BRUCELLA ABORTUS BIOVAR 1 STR- 9-941	ZZZZZZZZZZZZZZZZZZZZZZ
BRADYRHIZOBIUM SP- ORS278	ZZZZZZZZZZZZZZZZZZZZZZ
BRADYRHIZOBIUM SP- BTAI1	ZZZZZZZZZZZZZZZZZZZZZZ
BRADYRHIZOBIUM JAPONICUM USDA 110	ZZZZZZZZZZZZZZZZZZZZZZ
BEIJERINCKIA INDICA SUBSP- INDICA ATCC 9039	ZZZZZZZZZZZZZZZZZZZZZZ
BARTONELLA TRIBOCORUM CIP 105476	ZZZZZZZZZZZZZZZZZZZZZZ
BARTONELLA QUINTANA STR- TOULOUSE	ZZZZZZZZZZZZZZZZZZZZZZ
BARTONELLA HENSELAE	ZZZZZZZZZZZZZZZZZZZZZZ
BARTONELLA GRAHAMII AS4AUP	ZZZZZZZZZZZZZZZZZZZZZZ
BARTONELLA BACILLIFORMIS KC583	ZZZZZZZZZZZZZZZZZZZZZZ
AZORHIZOBIUM CAULINODANS ORS 571	ZZZZZZZZZZZZZZZZZZZZZZ
AURANTIMONAS SP- SI85-9A1	ZZZZZZZZZZZZZZZZZZZZZZ
AGROBACTERIUM VITIS S4	ZZZZZZZZZZZZZZZZZZZZZZ
AGROBACTERIUM TUMEFACIENS STR- C58	ZZZZZZZZZZZZZZZZZZZZZZ
AGROBACTERIUM RADIOBACTER K84	ZZZZZZZZZZZZZZZZZZZZZZ

[1] Clade classifications after leaving one tRNA functional class out from MLP multiway classifier model. Legend K: Rickettsiales, D: Rhodospirillales, S: Sphingomonadales, C: Caulobacterales, B: Rhodobacteraceae, H: Hyphomonadaceae, Z: Rhizobiales. For each genome, the 22 clade classifications are givenin order of decreasing robustness of classifications to deletion over all genomes considered “known” (all but SAR11, Stappia, Labrenzia and Pseudovibrio): F,T,K,E,L,X,P (203 out of 203 genomes), S (202 genomes), A,I (201 genomes), N,Y,Q,M,J,W (200 genomes) V,D (199 genomes), C,H,R,G (197 genomes).
