# Supplementary material for: tRNA Signatures Reveal a Polyphyletic Origin of SAR11 Strains among Alphaproteobacteria
Source: PLoS Comput Biol. 2014 Feb 27;10(2):e1003454. doi: 10.1371/journal.pcbi.1003454 (PMC3937112; doi:10.1371/journal.pcbi.1003454)
Supplement: Dataset S1 — Source code and data to reproduce Figure 2 . (ZIP) [file pcbi.1003454.s001.zip › Source_data_and_methods_for_Figure_2/tRNAlogofun.zip!/tRNAlogofun/tpro/tpro.html]

tpro -- make tRNA identity profiles


- NAME
- SYNOPSIS
- DESCRIPTION

- Sequence Input Format
- tpro Output

- OPTIONS
- PREREQUISITES
- AUTHOR
- SEE ALSO
- COPYRIGHT
- LICENSE


---


---

# NAME

tpro -- make tRNA identity profiles

---

# SYNOPSIS

To make tRNA logos of a set of tRNAs:

1. First align them:

Execute ``covea trna-prok2.cm tRNAdata.fas > tRNA.covea''

2. Then make profile matrices from the alignment:

```
Execute  "tpro tRNA.covea > data.mat"
```

3. Then make a function logo from the matrices:

Execute ``logofun --function data.mat''

---

# DESCRIPTION

## Sequence Input Format

tpro expects its query tDNA sequence input file to be a multi-fasta
file of unaligned tDNA sequences. The input sequences may be mixed
upper- and lowercase, but they should contain the character 'T'
instead of 'U'. Sequence characters other than {A,C,G,T,a,c,g,t} are
ignored. They do not contribute to the models nor the scores of data
sequences. Such ``bad'' characters are reported in program output, for
models in a file called MODEL.log.

tpro expects the identifiers of input sequences to be of one of a set
of currently three allowed formats. tfam uses this information to
recognize the annotated type of each tRNA to compare to its own
classification. The easiest way to make legal input sequence files
for tfam is to either generate them from the Sprinzl database with the
'sprinzl2fas' script, from tRNAscan-SE structural output files in
combination with genomic data using the 'tSE2fas' script, or by
directly feeding in a fasta file saved from the Genomic tRNA Database
(GtRDB, see website below). tSE2fas and sprinzl2fas are provided along
with tfam in the TFAM package or at the website given below. A short
description of these two legal input formats are as follows:

The translated Sprinzl identifier format is:

>[D|R][A-Z]nnnn\_XXX

where [D|R] means either ``D'' or ``R'' (DNA or RNA), [A-Z] means the
annotated type of tRNA (1-letter aa code, with 'X' as initiator), and
nnnn\_XXX means the Sprinzl taxonomic id (an integer of length 4)
followed by an underscore '\_' and a string of three characters
specifying the anticodon in a DNA alphabet. See documentation for
sprinzl2fas for more information. An example of a translated Sprinzl
identifier is:

>DH1000\_GTG

which means ``His-tDNA from taxon id 1000 with anticodon GTG''.

The translated tRNAscan-SE format is as follows:

>Yyyy[A-Z?$#]XXXggggggg

Where Yyyy is a taxonomic ID for the genome analyzed (one capital
letter followed by three of any character), [A-Z?$#] is a
one-character code for type of tRNA (either a one-letter aa code, 'X'
for initiator, ? for 'undetermined', '$' for 'pseudo-tRNA', or '#' for
'unalignable' or 'UAR anticodon'), 'XXX' is an anticodon string in the
DNA alphabet, and 'gggggg' is an arbitrary-length string of integers
indicating the genome location of the tDNA. See documentation for
tSE2fas for more information. An example of a translated Sprinzl
identifier is:

>BsisHGTG-3171484

which means ``His-tDNA from taxon id Bsis with anticodon GTG and at
position -3171484'' (the negative refers to strand and can be ignored).

Support for tpro to parse tfam output and the GtRDB format is provided
but not recently tested.

tpro only uses information about the type of amino acid from the
identifier. If you wish for another identifier format to be supported,
or have problems, please contact me.

## tpro Output

Currently tfam will overwrite existing output files. So be careful and
run tfam in a fresh directory!

---

# OPTIONS

**-h** Print short usage information and quit


**-o** Print Academic Free License Conditions for Using, Copying and Redistribution of tfam

```
=item *
```

**-x \_positions\_** Exclude positions \_positions\_. \_positions\_ is a sequence of comma-separated ranges of digits referring to columns in a coveaf output file from a prior run. The offset is 1, so the first column is position '1'. Example: '1,33-36,45'

---

# PREREQUISITES

tpro requires bioperl, and PDL

---

# AUTHOR

David H. Ardell, for email visit http://www.lcb.uu.se/~dave

---

# SEE ALSO

TFAM website : http://www.lcb.uu.se/~dave/TFAM


COVEMF website : http://www.lcb.uu.se/~dave/COVEMF


GtRDB website : http://rna.wustl.edu/GtRDB


Sprinzl DB website : http://www.uni-bayreuth.de/departments/biochemie/sprinzl/trna/

---

# COPYRIGHT

tpro is Copyright (C) 2004,
by David H. Ardell. All rights reserved. This is free software; see
the source for copying conditions. There is NO warranty; not even for
MERCHANTABILITY or FITNESS FOR A PARTICULAR PURPOSE.

---

# LICENSE

Distributed under the terms of the GNU GENERAL PUBLIC LICENSE Version
2. Please see http://www.gnu.org/licenses/gpl.html or the source
for the text of this license.
